# Identifying optimal candidates for post-TIPS patients with HCC undergoing TACE: a multicenter observational study

**DOI:** 10.1007/s00330-022-09249-6

**Published:** 2022-12-23

**Authors:** Wenzhe Fan, Bowen Zhu, Shufan Yue, Xinlin Zheng, Guosheng Yuan, Lei Yu, Wanchang Huang, Shugui Huang, Wenjiang Wei, Fuliang Li, Zhen Huang, Rong Tang, Huishuang Fan, Zhuoyong Li, Liangliang Qiao, Fuxi Huang, Yu Cheng, Yingqiang Zhang, Yanqin Wu, Xinhua Zou, Miao Xue, Hongyu Wang, Jiaping Li

**Affiliations:** 1 Department of Interventional Oncology, The First Affiliated Hospital of Sun Yat-sen University, 58 Zhongshan 2nd Road, Guangzhou, 510080 People’s Republic of China; 2 Department of Ultrasonic, The First Affiliated Hospital of Sun Yat-sen University, Guangzhou, People’s Republic of China; 3 Department of Gastroenterology, Nanfang Hospital, Guangzhou, People’s Republic of China; 4 Department of Interventional Radiology, The People’s Hospital of Guangxi Zhuang Autonomous Region, Nanning, Guangxi People’s Republic of China; 5 Department of Interventional Radiology, The First People’s Hospital of Yulin, Yulin, Guangxi People’s Republic of China; 6 Department of Intervention, The First Affiliated Hospital of Guangzhou Pharmaceutical University, Guangzhou, People’s Republic of China; 7 Department of Intervention, Guangdong Second Provincial General Hospital, Guangzhou, People’s Republic of China; 8 Liver and Gall Surgical Department, Gaozhou People’s Hospital, Gaozhou, People’s Republic of China; 9 Interventional Vascular Department, Huizhou First Hospital, Huizhou, People’s Republic of China; 10 Department of Liver and Gallbladder Surgery, Hainan General Hospital, Haikou, People’s Republic of China; 11 Interventional Department, Dongguan People’s Hospital, Dongguan, People’s Republic of China; 12 Department of Radiology, Jiangmen Central Hospital, Jiangmen, People’s Republic of China; 13 Department of Interventional Oncology, Jinshazhou Hospital of Guangzhou University of Chinese Medicine, Guangzhou, People’s Republic of China; 14 Department of Oncology, Guangzhou Panyu Central Hospital, Guangzhou, People’s Republic of China; 15 Department of Liver and Gallbladder Surgery, Huizhou Central Hospital, Huizhou, People’s Republic of China; 16 Interventional Department, The Seventh Affiliated Hospital of Sun Yat-sen University, Shenzheng, People’s Republic of China

**Keywords:** Prognosis, Risk stratification, Transjugular intrahepatic portosystemic shunt, Hepatocellular carcinoma, Transarterial chemoembolization

## Abstract

**Objective:**

To develop a prognostic model for post-transjugular intrahepatic portosystemic shunt (TIPS) patients with hepatocellular carcinoma (HCC) beyond the Milan criteria treated by transarterial chemoembolization (TACE).

**Design:**

Between January 2013 and January 2020, 512 patients with HCC beyond the Milan criteria who underwent TACE after TIPS were retrospectively recruited from 15 tertiary centers. Patients were randomly sorted into a training set (*n* = 382) and a validation set (*n* = 130). Medical data and overall survival were assessed. A prediction model was developed using multivariate Cox regression analyses. Predictive performance and discrimination were evaluated and compared with other prognostic models.

**Results:**

Vascular invasion, log_10_(AFP), 1/creatinine, extrahepatic spread, and log_10_(ALT) were the most significant prognostic factors of survival. These five parameters were included in a new VACEA score. This score was able to stratify patients in the training set into four distinct risk grades whose median overall survival were 25.2, 15.1, 8.9, and 6.2 months, respectively. The 6-month, 1-year, 2-year, and 3-year AUROC values and C-index of the VACEA model were 0.819, 0.806, 0.779, 0.825, and 0.735, respectively, and higher than those of other seven currently available models in both the training and validation sets, as well as in different subgroups.

**Conclusion:**

The VACEA score could stratify post-TIPS patients with HCC beyond the Milan criteria treated by TACE and help to identify candidates who benefit from this treatment.

**Key Points:**

*• Vascular invasion, AFP, creatinine, extrahepatic spread, and ALT were independent significant prognostic factors of survival for HCC patients who underwent TACE after TIPS.*

*• Our new model, named VACEA score, can accurately predict prognosis at the individual level and stratify patients into four distinct risk grades.*

*• The VACEA model showed better prognostic discrimination and calibration than other current TACE-/TIPS-specific models*

**Figure:**
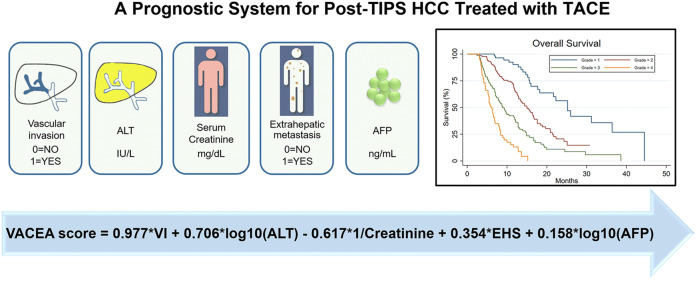
Graphical abstract

**Supplementary Information:**

The online version contains supplementary material available at 10.1007/s00330-022-09249-6.

## Introduction

In patients with hepatocellular carcinoma (HCC) within the Milan criteria (one lesion up to 5 cm or ≤ 3 lesions over 3 cm, without vascular invasion or extrahepatic metastasis) and with decompensated cirrhosis, hepatic transplantation is the first-line therapy [1]. Nevertheless, > 70% of patients with HCC in China have a tumor burden beyond the Milan criteria at the time of diagnosis and lose the chance for a liver transplant [2]. In these patients, liver cirrhosis and portal vein invasion favor portal hypertension and potential variceal bleeding and/or refractory ascites [3]. Transjugular intrahepatic portosystemic shunt (TIPS) is considered a safe and effective strategy for managing portal hypertension, creating opportunities for tumor treatment to HCC [4, 5]. However, at present, there are no treatment guidelines for patients with HCC beyond the Milan criteria after TIPS insertion.

Recommended for inoperable HCC > 5 cm [6], transarterial chemoembolization (TACE) has a high objective response rate (ORR) and is an effective option for unresectable HCC [7]. TACE has been used for post-TIPS patients with HCC [8, 9]. However, hepatic artery embolization may further reduce hepatic perfusion in patients who have undergone TIPS because a patent shunt diverts portal blood flow away from the liver; therefore, TACE might not be ideal for patients with HCC post-TIPS [4, 10]. TACE is potentially indicated for patients with well-preserved liver function, and only if a super-selective hepatic arterial embolization is possible, or, in very selected cases, as a bridge to liver transplantation [8].

According to previous studies, survival outcome of post-TIPS TACE is highly heterogeneous (Supplemental Table 1). In patients with Barcelona Clinic Liver Cancer (BCLC) stage B or C HCC, repeated TACE can be safely performed in selected post-TIPS patients with a survival benefit [11]. However, there is a high 1 month incidence (36.0%) of severe adverse events of hepatotoxicity (grade ≥ 3 ) after TACE in post-TIPS patients with higher tumor burdens [4]. In addition, the local efficacy of TACE is worse in patients who underwent TIPS than in those who did not [9].

There are several prognostic models for patients with unresectable HCC treated with TACE, such as the Pre-TACE-Predict model [12], HAP score [13], mHAP-II score [14], and mHAP-III score [15]. In addition, rating system for the liver function of patients with HCC includes the albumin-bilirubin (ALBI) score [16], and risk score for patients undergone TIPS, includes the Model for End-stage Liver Disease (MELD) [17] and Freiburg index of post-TIPS survival (FIPS) [18] (Supplemental Table 2). However, none is consistent for patients who received TACE after TIPS. Although we previously reported the safety and efficacy of TACE in the treatment of HCC patients after TIPS [19], further identification of patients who may benefit from this therapy is warranted. Therefore, the aim of this study was to develop an alternative model that can be used to predict survival in patients with HCC beyond the Milan criteria who were treated with TACE after TIPS, and help to identify the ideal candidates.

## Materials and methods

### Study population

This retrospective study collected data from 15 tertiary medical centers from January 2013 to January 2020. Approval was obtained from the institutional review board of Sun Yat-Sen University First Affiliated Hospital (Approval ID 2022[053]), and informed consent was waived because of the study’s retrospective design. This analysis was reported according to the Transparent Reporting of a Multivariable Prediction Model for Individual Prognosis or Diagnosis (TRIPOD) guidelines [20].

The eligibility criteria were (a) age 18–75 years; (b) diagnosis of HCC according to the American Association for Liver Disease and European/American Association for Liver Disease guidelines [21, 22]; (c) tumor burden beyond the Milan criteria; (d) history of undergoing TIPS as a secondary preventive measure for variceal bleeding or refractory ascites; (e) TACE was the first line treatment to HCC after the patients with TIPS and patent portal vein vascular perfusion exhibited throughout the stent with a mid-stent Doppler velocity > 60 cm/s [23]; (f) Eastern Cooperative Oncology Group performance (ECOG) status score of 0 or 1; and (g) Child-Pugh A–B class.

The exclusion criteria were (a) portal vein tumor thrombus (PVTT) in the main portal vein; (b) history of liver transplantation after TIPS; (c) severe dysfunction of the heart, kidney, or other organs; (d) history of a secondary malignancy; and (e) contraindication for TACE because of severe coagulation disorders and hepatic encephalopathy.

Patients within each center were randomly assigned to training or validation datasets at a 3:1 ratio according to computer-generated randomized numbers.

### TACE procedures

TACE included conventional TACE and drug-eluting bead TACE. Details are shown in supplemental method.

### Outcomes assessment

The OS was defined as the period from the first TACE after TIPS until death or last follow-up. All patients underwent triphasic contrast-enhanced computed tomography (CT) or magnetic resonance imaging (MRI). Serum AFP, alanine transaminase (ALT), and aspartate aminotransferase (AST) levels were assessed within 72 h before TACE. Tumor response and safety were assessed at 4–6-week intervals until death or last follow-up. CT or MRI images were used to assess the efficacy of local tumor response according to modified Response Evaluation Criteria in Solid Tumors (mRECIST) criteria [24]. ORR was defined as the sum of complete response and partial response. The best overall response during treatment was considered the final response. On-demand TACE procedures were scheduled at an interval of 6–12 weeks upon demonstration of viable tumors or intrahepatic recurrences by CT/MRI in patients with the same clinical and laboratory findings (e.g., performance status, liver function). The last follow-up date was September 1, 2021.

### Statistical analysis

Survival curves were estimated using the Kaplan-Meier analyses and compared by log-rank test. Univariate Cox regression analyses were applied to the training cohort to identify prognostic factors. Variables with *p* values < 0.05 in univariate analysis were included in multivariate analysis. A multivariate Cox proportional hazards model was used to identify the independent risk factors associated with OS. The newly developed scoring system was based on the abovementioned analyses and was named VACEA score (taken from the initials of VI, ALT, creatinine, EHS, AFP). Discrimination and performance were measured by Harrell’s C concordance index (C-index), likelihood ratio chi-square, and area under the time-dependent receiving operator characteristic curve, respectively. Calibration was assessed by splitting the new score into quintiles and comparing the observed and predicted 12-month survival rate, as well as by visual inspection of Kaplan-Meier curves. The VACEA score was compared with prognostic models, including the Pre-TACE-Predict model [12], HAP score [13], modified HAP-II (mHAP-II) score [14], modified HAP-III (mHAP-III) score [15], albumin-bilirubin (ALBI) score [16], MELD score [17], and FIPS score [18] in both training and validation datasets. All statistical tests were two-sided, and a *p* value < 0.05 indicated statistical significance. All statistical analyses were performed using R version 4.0.2 and STATA version 15.0 (StataCorp Lp).

## Results

### Baseline characteristics

**Fig. 1:**
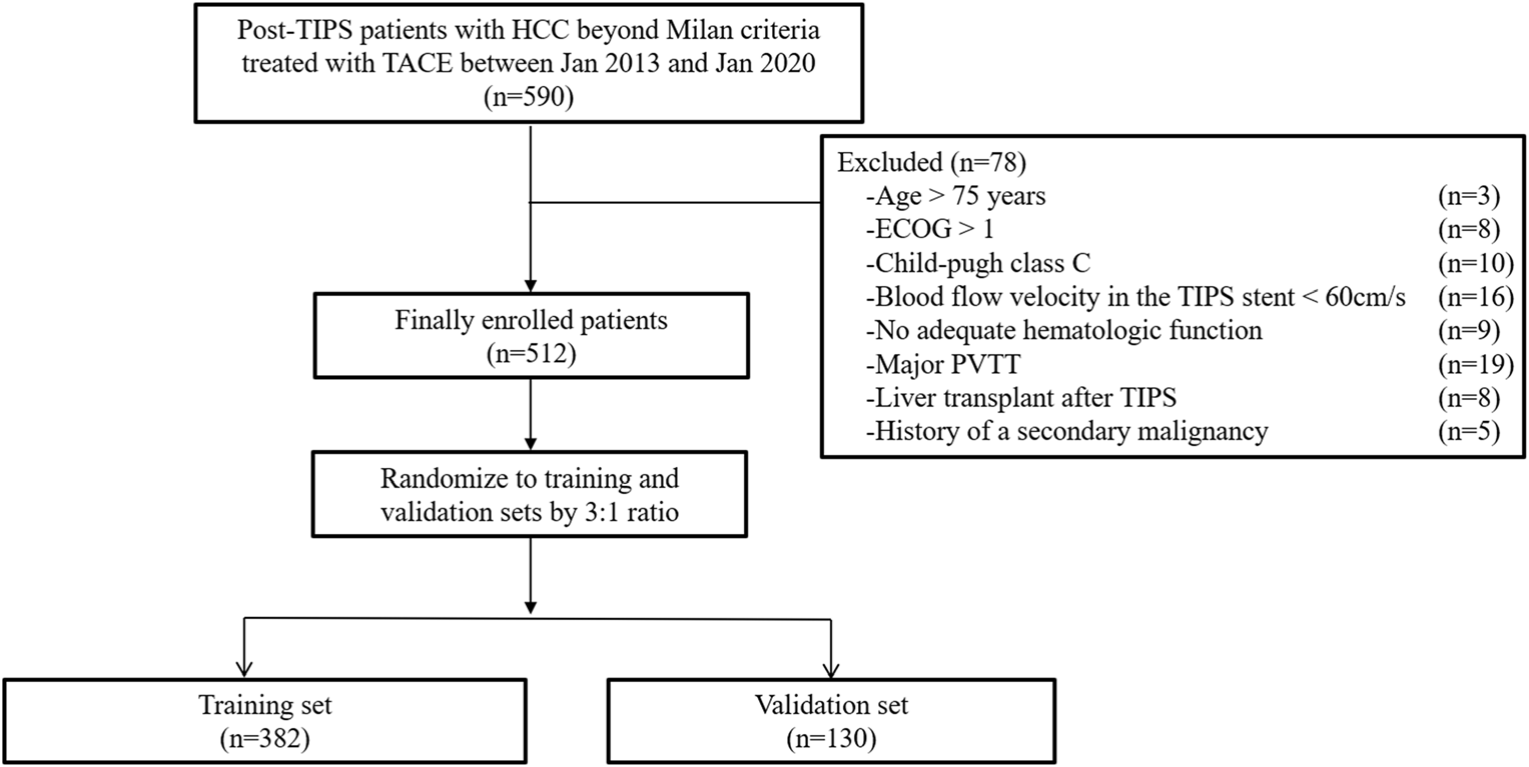
Flowchart depicting the patient selection process. ECOG, Eastern Cooperative Oncology Group; HCC, hepatocellular carcinoma; PVTT, portal vein tumor thrombus; TACE, transarterial chemoembolization; TIPS, transjugular intrahepatic portosystemic shunt

A total of 512 patients with HCC beyond the Milan criteria who underwent TACE after TIPS between January 2013 and January 2020 were enrolled in this retrospective study and randomly sorted into the training (*n* = 382) and validation (*n* = 130) datasets (shown in Fig. 1). The median time between TIPS and the first TACE in this cohort of patents having HCC combined with portal hypertension complications when first diagnosis was 11 (range: 7–26) days, and the mean time interval was 12.8 days. There were no differences in baseline demographics between datasets (Table 1). The baseline characteristics of patients from each institute are shown in Supplemental Table 3.

**Table 1:** Characteristics of patients in the training and validation sets

Baseline characteristics	Number (%)/median (quartile)		*p* value
	Training set (*n* = 382)	Validation set (*n* = 130)	
Age (years)	52 (42–60)	53 (43–60)	0.603
< 50	155 (40.6)	51 (39.2)	
≥ 50	227 (59.4)	79 (60.8)	
Sex			0.976
Male	350 (91.6)	119 (91.5)	
Female	32 (8.4)	11 (8.5)	
Etiology			0.664
HBV	337 (88.2)	118 (90.8)	
HCV	12 (3.1)	4 (3.1)	
Other	33 (8.6)	8 (6.2)	
TIPS indication			0.820
Secondary prevention of variceal bleeding	308 (80.6)	106 (81.5)	
Ascites	74 (19.4)	24 (18.5)	
ECOG score			0.654
0	281 (73.6)	93 (71.5)	
1	101 (26.4)	37 (28.5)	
Platelet count (×10^9^/L)	138 (97–206)	137 (96–193)	0.299
AFP (ng/mL)	315.9 (26.5–7162)	419.8 (30.5–8340)	0.670
< 400	199 (52.1)	62 (47.7)	
≥ 400	183 (47.9)	68 (52.3)	
ALT (IU/L)	36 (23–56)	34 (22–57)	0.427
AST (IU/L)	55 (34–89)	56 (35–89)	0.458
ALB (g/L)	35.3 (31.3–38.8)	35.5 (32.0–38.9)	0.303
TBil (μmol/L)	18.2 (13.2–27.1)	18.6 (12.8–27.1)	0.846
Ammonia (μmol/L)	65 (39–90)	66 (40–90)	0.710
Creatinine (mg/dL)	0.85 (0.72–1.01)	0.85 (0.71–1.02)	0.726
INR	1.14 (1.05–1.24)	1.13 (1.02–1.25)	0.998
Child-Pugh class			0.443
A	287 (75.1)	102 (78.5)	
B	95 (24.9)	28 (21.5)	
Intrahepatic tumors number			
Single	67 (17.5)	20 (15.4)	0.572
Multiple	315 (82.5)	110 (84.6)	
Main tumor size (cm)	7.8 (4.0–10.8)	7.8 (4.1–10.6)	0.883
Vascular invasion			0.417
No	129 (33.8)	49 (37.7)	
Yes	253 (66.2)	81 (62.3)	
Extrahepatic spread			0.603
No	258 (67.5)	91 (70.0)	
Yes	124 (32.5)	39 (30.0)	
BCLC stage			0.245
A	24 (6.3)	6 (4.6)	
B	93 (24.3)	41 (31.5)	
C	265 (69.4)	83 (63.8)	
ALBI score	−2.15 (−2.52– [−1.76])	−2.14 (−2.53– [−1.85])	0.358
FIPS score	0.97 (0.53–1.34)	0.971 (0.49–1.38)	0.971
MELD score	10.24 (9.76–10.73)	10.22 (9.83–10.66)	0.767
Pre-TACE-Predict score	2.31 (1.73–2.82)	2.31 (1.79–2.89)	0.840
HAP score	2 (1–3)	2 (1–3)	0.570
mHAP-II score	3 (2–4)	3 (2–4)	0.477
mHAP-III score	−9.75 (−11.75– [−7.37])	−10.00 (−11.66– [−7.80])	0.622

*AFP*, alpha-fetoprotein; *ALB*, albumin; *ALT*, alanine aminotransferase; *AST*, aspartate aminotransferase; *BCLC*, Barcelona Clinic Liver Cancer; *CI*, confidential interval; *HBV*, hepatitis B virus; *PLT*, platelet count; *PVTT*, portal vein tumor thrombus; *RBC*, red blood cells; *TBil*, total bilirubin; *WBC*, white blood cells; *INR*, international normalized ratio

### Treatment outcome

The median survival of the entire cohort was 12.5 (95% CI: 11.7-13.4) months, with 6-month, 1-year, 2-year, and 3-year survival rates being 80.2%, 51.7%, 20.8%, and 13.2%, respectively (shown in Supplemental Fig. 1A). There was no difference in the median survival between the training (12.7 [95% CI: 11.7-13.7] months) and validation datasets (11.9 [95% CI: 9.9-13.9] months; *p* = 0.710; shown in Supplemental Fig. 1B). ORR according to mRECIST criteria of the entire cohort, the training, and validation datasets are 64.1%, 64.9%, and 61.5%, respectively (Supplemental Table 4).

### Univariate and multivariate analysis

The results of univariate and multivariate analyses are presented in Table 2. Univariate analysis showed log_10_(tumor size), log_10_(AFP), log_10_(bilirubin), log_10_(ALT), log_10_(AST), 1/creatinine, vascular invasion (VI), and extrahepatic spread (EHS) were significantly correlated with OS. Multivariate Cox proportional hazards analysis showed that VI (HR = 2.637, *p* < 0.001), EHS (HR = 1.415, *p* = 0.021), log_10_(AFP) (HR = 1.139, *p* = 0.003), log_10_(ALT) (HR = 1.980, *p* = 0.018), and 1/creatinine (HR = 0.529, *p* = 0.021) were independent factors for OS.

**Table 2:** Univariate and multivariate analyses of overall survival predictors

Factor	Univariate				Multivariate			
	β	HR	95% CI	*p* value	β	HR	95% CI	*p* value
Age (years)	−0.011	0.989	0.977–1.000	0.054				
Sex								
Female		Ref						
Male	0.201	1.222	0.724–2.06	0.453				
Etiology								
Other		Ref						
HBV	0.235	1.264	0.821–1.946	0.286				
TIPS indication								
Secondary prophylaxis of variceal bleeding		Ref						
Ascites	−0.128	0.880	0.638–1.213	0.434				
ECOG score								
0		Ref						
1	0.285	1.33	1.002	1.766				
Tumor number								
Single		Ref						
Multiple	0.271	1.312	0.907–1.897	0.149				
Log_10_ Main tumor size	0.844	2.326	1.478–3.662	< 0.001	0.393	1.481	0.949–2.311	0.084
Vascular invasion								
No		Ref				Ref		
Yes	1.206	3.339	2.463	< 0.001	0.970	2.637	1.892–3.677	< 0.001
Extrahepatic spread								
No		Ref				Ref		
Yes	0.812	2.253	1.720–2.951	< 0.001	0.347	1.415	1.055–1.897	0.021
Log_10_ AFP	0.251	1.286	1.190–1.390	< 0.001	0.130	1.139	1.044–1.24	0.003
Log_10_ Ammonia	−0.001	0.999	0.994–1.003	0.671				
Log_10_ Albumin	−1.513	0.220	0.032–1.531	0.126				
Log_10_ bilirubin	0.647	1.910	1.210–3.015	0.005	−0.023	0.977	0.583–1.637	0.930
Log_10_ ALT	0.763	2.146	1.493–3.084	< 0.001	0.683	1.980	1.123–3.493	0.018
Log_10_ AST	0.862	2.368	1.657–3.384	< 0.001	0.132	1.141	0.607–2.147	0.682
1/creatinine	−0.832	0.435	0.261–0.726	0.001	−0.638	0.529	0.308–0.909	0.021
1/INR	−0.905	0.405	0.131–1.248	0.115				
Platelets	0.000	1.000	0.998–1.001	0.545				
Child-Pugh class								
A		Ref						
B	−0.177	0.837	0.625–1.122	0.235				

Note: *Tumor size*, size of the largest tumor; *AFP*, alpha-fetoprotein; *ECOG*, Eastern Cooperative Oncology Group; *HBV*, hepatitis B virus; *PLT*, platelet count; *PVTT*, portal vein tumor thrombus; *TBil*, total bilirubin; *INR*, international normalized ratio

### Development of the prognostic model

The abovementioned five variables were used to develop the final prognostic model; β-coefficients are shown in Table 3. Using the regression coefficients of the multivariable model, the linear predictor was calculated as follows: linear predictor = 0.977*VI (0 = no, 1 = yes) + 0.706*log_10_(ALT) (IU/L) − 0.617*1/creatinine (mg/dL) + 0.354*EHS (0 = no, 1 = yes) + 0.158*log_10_(AFP) (ng/mL). This calculated linear predictor represents the new prognostic model for patients with HCC beyond the Milan criteria who underwent TACE after TIPS and was named VACEA.

**Table 3:** Prognostic factors and estimated scores in the training set

Variable	β	HR	95% CI	*p-*value
Vascular invasion				
No		Ref		
Yes	0.977	2.655	1.909–3.694	< 0.001
Extrahepatic spread				
No		Ref		
Yes	0.354	1.424	1.064–1.907	0.018
Log_10_ AFP	0.158	1.171	1.078–1.272	< 0.001
Log_10_ ALT	0.706	2.025	1.363–3.009	< 0.001
1/Creatinine	−0.617	0.539	0.315–0.923	0.024

Note: *AFP*, alpha-fetoprotein; *ALT*, alanine aminotransferase

**Fig. 2:**
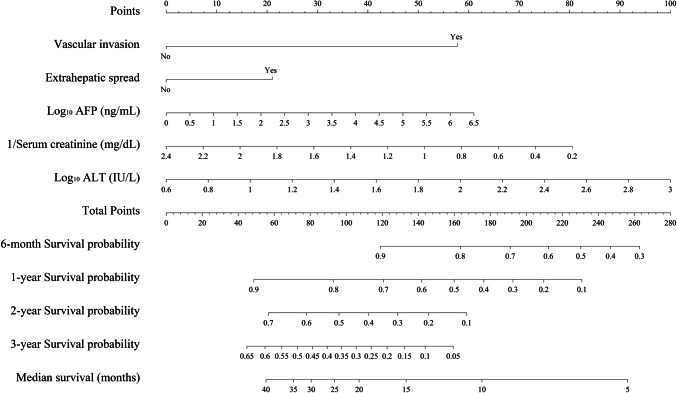
Nomogram of the VACEA model for individual survival prediction. AFP, alpha-fetoprotein; ALT, alanine aminotransferase

Survival probability at t months for a given patient was calculated as follows: S(t) = S_0_(t)exp(score-1.657). S_0_(t) represents the survival probability for a patient with the mean VACEA score (= 1.657). S_0_(t) is 0.825, 0.531, 0.250, and 0.153 for survival probability at 6, 12, 18, and 24 months, respectively. A nomogram for individual patient risk stratification was created to predict the 6-month, 1-year, 2-year, and 3-year survival probability and estimated median survival (shown in Fig. 2). In addition, patient prognosis at 6, 12, 18, and 24 months can be assessed using an online calculator (https://jscalc.io/calc/bS6XkBa4aTyigfmD).

### The VACEA model predicts overall survival

**Fig. 3:**
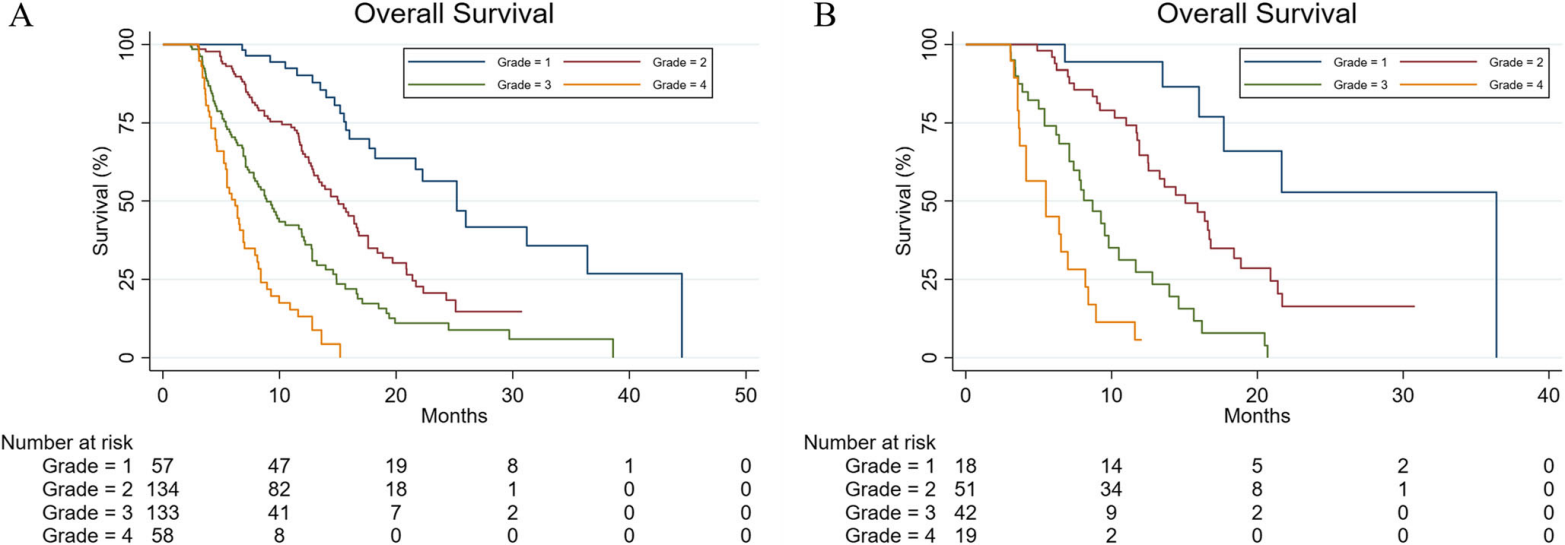
Overall survival according to risk grades as defined by the VACEA score in the two cohorts. Overall survival in the (**A**) training and (**B**) validation sets

To generate four risk grades, the following cutoffs were applied (determined by the fifteenth, fiftieth, and eighty-fifth centiles in the training set): ≤ 0.604 (grade 1), > 0.604 to ≤ 1.657 (grade 2), > 1.657 to ≤ 2.343 (grade 3), and > 2.343 (grade 4) (shown in Fig. 3). The median patient OS in the four grades were 25.2, 15.1, 8.9, and 6.2 months in the training set, and 36.4, 15.1, 8.7, and 5.5 months in the validation set. The 6-month, 1-year, and 2-year survival rates of all grades in the training and validation sets are shown in Supplemental Table 5. The ORR of the four grades were 93.0%, 74.6%, 53.4%, and 41.4% in the training set, and 94.4%, 70.6%, 52.4%, and 26.3% in the validation set (Supplemental Table 6). Survival curves and tumor responses were significantly different among the four risk grades in the training and validation sets.

### Discrimination and calibration of the VACEA model and comparison with other models

**Fig. 4:**
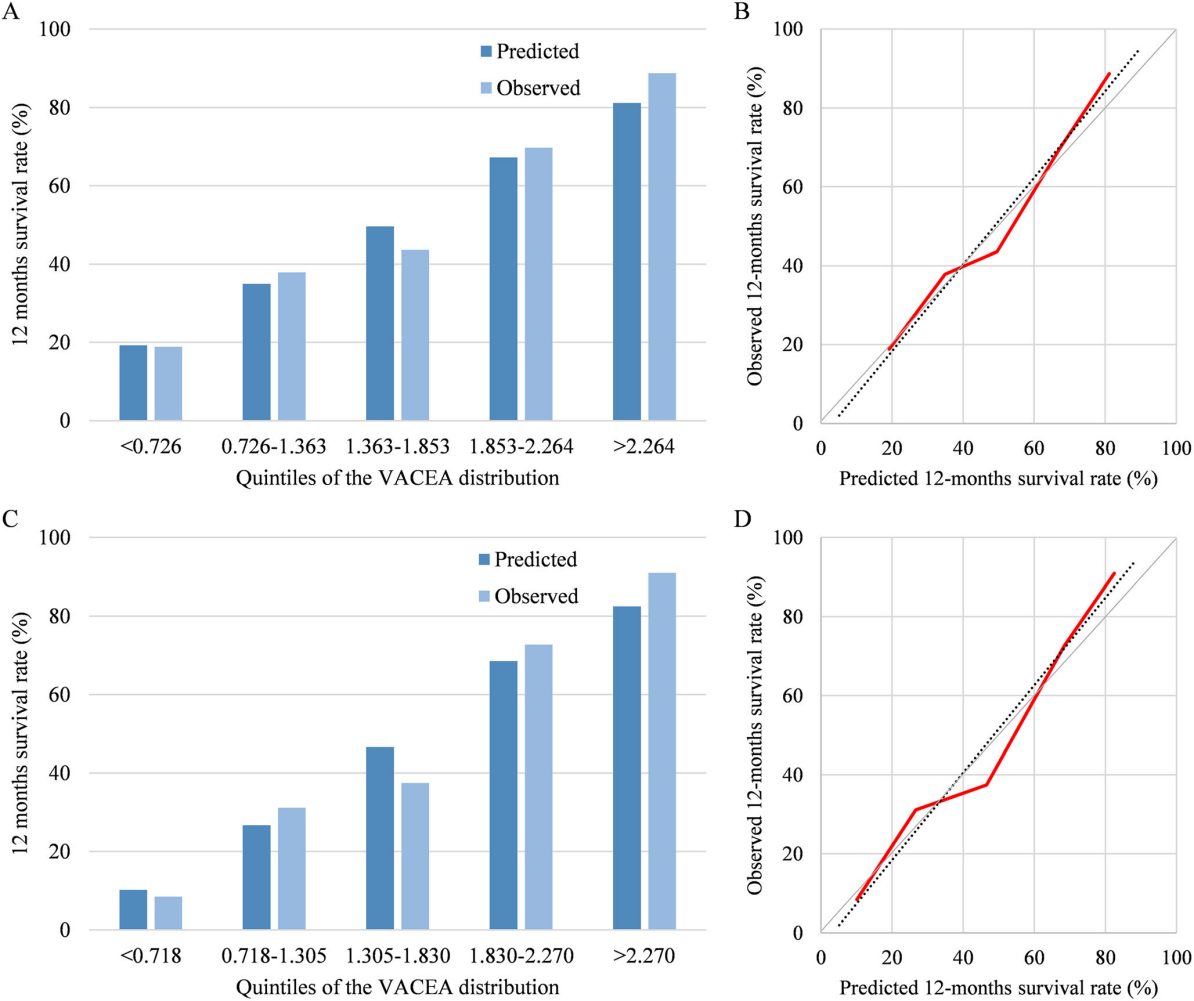
Calibration of 12-month survival of the VACEA model. **A** Observed and predicted 12 months survival rate in the training set. **B** Calibration curves of the VACEA model in the training set (red line), slope of straight-line least squares fit to calibration (dashed line) was 1.100. **C** Observed and predicted 12-month survival rate in the validation set. **D** Calibration curves of the VACEA model in the validation set (red line), slope of straight-line least squares fit to calibration (dashed line) was 1.105

The discrimination of the current model was measured by the likelihood ratio *χ*^2^, C-index, and Akaike information criterion, which showed a good performance of the VACEA model in the training and validation datasets (Supplemental Table 7). The Hosmer-Lemeshow test showed similar observed and predicted 12-month survival rate of the VACEA score in the training set (*χ*^2^ = 9.238, *p* = 0.323, slope of calibration curve = 1.100; shown in Fig. 4A, B) and validation set (*χ*^2^ = 12.647, *p* = 0.125, slope of calibration curve = 1.105; shown in Fig. 4C, D). Moreover, the Kaplan-Meier curves comparing observed vs. predicted survival showed good calibration of the VACEA score at different risk grades (shown in Supplemental Fig. 2).

**Fig. 5:**
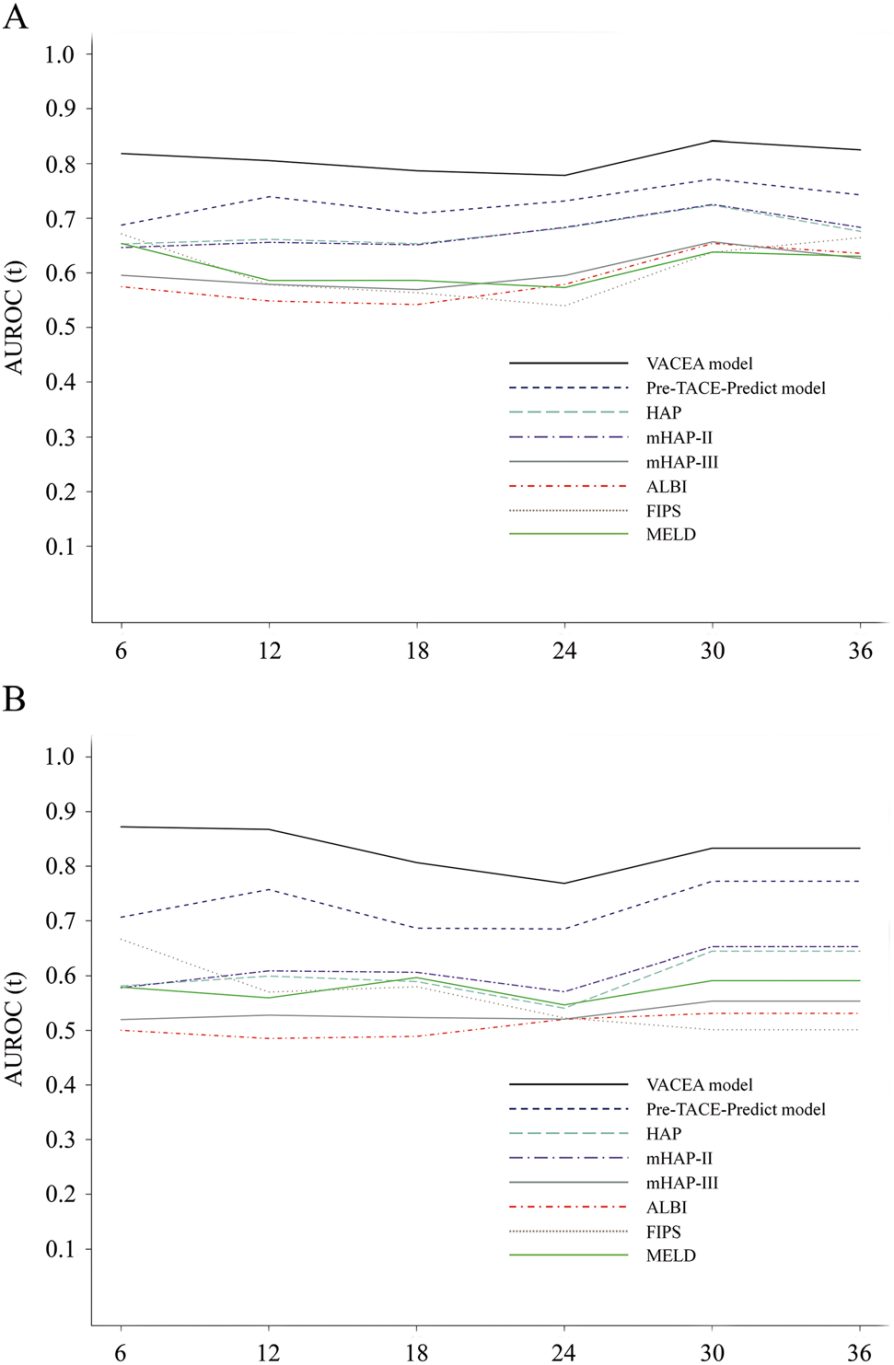
Time-dependent AUROC values of the VACEA model and other models. **A** Time-dependent AUROC values in the training set; **B** time-dependent AUROC values in the validation set. AUROC, area under receiver operating characteristic curve

The performance of the VACEA model and the other models (Pre-TACE-Predict model, FIPS model, MELD score, ALBI score, HAP score, mHAP-II score, and mHAP-III score) was compared using the area under receiver operating characteristic curve (AUROC) and C-index. The 6-month, 1-year, 2-year, and 3-year AUROC values and C-index of the VACEA model were higher than those of the other models (shown in Fig. 5, Table 4), suggesting a favorable performance and discrimination of our model. Similar results were obtained in age, AFP levels, ECOG score, etiology, and TIPS indications subgroups (Supplemental Table S8-9).

**Table 4:** Comparison of the performance and discriminative ability between the VACEA model and other models

Cohort	Models	6-month AUROC (95% CI)	1-year AUROC (95% CI)	2-year AUROC (95% CI)	3-year AUROC (95% CI)	C-index (95% CI)
Training	VACEA	0.819 (0.774–0.864)	0.806 (0.756–0.856)	0.779 (0.686–0.872)	0.825 (0.669–0.981)	0.735 (0.705–0.764)
	Pre-TACE-Predict	0.688 (0.626–0.750)	0.739 (0.682–0.796)	0.732 (0.612–0.852)	0.742 (0.521–0.963)	0.670 (0.635–0.706)
	FIPS	0.671 (0.600–0.742)	0.578 (0.513–0.643)	0.539 (0.423–0.655)	0.664 (0.411–0.917)	0.582 (0.541–0.623)
	MELD	0.654 (0.585–0.723)	0.586 (0.522–0.650)	0.573 (0.444–0.702)	0.630 (0.274–0.986)	0.587 (0.547–0.627)
	ALBI	0.575 (0.501–0.649)	0.548 (0.483–0.613)	0.579 (0.463–0.695)	0.635 (0.490–0.780)	0.538 (0.496–0.581)
	HAP	0.653 (0.587–0.719)	0.661 (0.602–0.720)	0.682 (0.562–0.802)	0.676 (0.468–0.884)	0.623 (0.585–0.662)
	mHAP-II	0.646 (0.580–0.712)	0.656 (0.596–0.716)	0.683 (0.564–0.802)	0.683 (0.459–0.907)	0.621 (0.583–0.659)
	mHAP-III	0.596 (0.525–0.667)	0.579 (0.515–0.643)	0.595 (0.469–0.721)	0.627 (0.385–0.869)	0.562 (0.521–0.603)
Validation	VACEA	0.872 (0.803–0.941)	0.867 (0.794–0.940)	0.768 (0.596–0.940)	0.833 (0.790–0.876)	0.771 (0.727–0.816)
	Pre-TACE-Predict	0.706 (0.600–0.812)	0.757 (0.661–0.853)	0.685 (0.483–0.887)	0.772 (0.692–0.852)	0.685 (0.628–0.743)
	FIPS	0.666 (0.536–0.796)	0.570 (0.459–0.681)	0.523 (0.324–0.722)	0.501 (0.150–0.852)	0.583 (0.514–0.653)
	MELD	0.579 (0.441–0.717)	0.560 (0.449–0.671)	0.547 (0.313–0.781)	0.591 (0.301–0.872)	0.566 (0.496–0.636)
	ALBI	0.500 (0.364–0.636)	0.485 (0.372–0.598)	0.520 (0.291–0.749)	0.531 (0.244–0.818)	0.492 (0.419–0.565)
	HAP	0.581 (0.453–0.709)	0.600 (0.494–0.706)	0.541 (0.339–0.743)	0.644 (0.335–0.953)	0.580 (0.511–0.649)
	mHAP-II	0.578 (0.458–0.698)	0.609 (0.503–0.715)	0.571 (0.377–0.765)	0.653 (0.335–0.971)	0.588 (0.524–0.652)
	mHAP-III	0.520 (0.391–0.649)	0.528 (0.415–0.641)	0.521 (0.297–0.745)	0.554 (0.207–0.901)	0.528 (0.458–0.597)

*AUROC*, area under the receiver operating characteristic curve; *ALBI*, albumin-bilirubin; *mHAP-II/III*, modified HAP-II/III; *FIPS*, Freiburg index of post-TIPS survival; *TACE*, transarterial chemoembolization; *TIPS*, transjugular intrahepatic portosystemic shunt; *MELD*, Model for end-stage liver disease

## Discussion

This study, based on a multicenter cohort with a sample size of 512 post-TIPS TACE candidates with HCC beyond the Milan criteria, attempted to establish a model that could predict survival probabilities on the basis of routine clinical features. VACEA score is the first model to stratify TACE-TIPS patient survival outcomes with a favorable performance and discrimination compared with the most frequently used current TACE or TIPS prognostic models, maybe helping to select the ideal post-TIPS TACE candidates.

The distinctive finding of this study is the establishment of an easy-to-use prognostic model for patients with HCC undergoing TACE after TIPS. The nomogram and online calculator can be easily applied for individual patient-level prognostication. This model provides consistent data for estimates of outcome in most scenarios of TACE for HCC patient post-TIPS, and identifies four risk grades. First, patients in grade 1 or grade 2 in our study had a median OS of 25.2 and 15.1 months, respectively, similar to OS (17 months) of patients with BCLC stage A or B HCC treated with TACE after TIPS reported [25]. That indicates patients in these two groups should be good candidates for TACE. Second, patients in grade 3 achieved a median OS of 8.9 months, similar to that of patients with BCLC stage C HCC treated with TACE after TIPS but still significantly longer than that of patients treated with sorafenib monotherapy [26]. In contrast, patients in grade 4 had no survival benefit of TACE with a median OS of 6.2 months. This OS was similar to that of patients with PVTT partial occlusion who underwent palliative treatment after TIPS (median OS 133 days) [27]. Therefore, only systemic therapy or palliative care is recommended in this category.

The survival outcome (ORR 64.1% and median OS 12.5 months) of this study was similar to that of post-TIPS patients with BCLC A-C stage HCC treated with TACE alone (ORR 65.4%, OS 14.0 months) [28]. This suggests that the present cohort is representative of the current clinical practice of TACE for post-TIPS patients with intermediate and advanced HCC. However, the median OS in our study is shorter than the reported 19.4 months in a systematic review on TACE-treated unresectable HCC [29], indicating that the prognosis of patients who underwent TACE with TIPS is impaired compared to those without.

AFP, VI, and EHS were negative prognostic factors associated with tumor burden of HCC patients. Secreted by ~70% of patients with HCC, AFP is a recognized tumor marker for HCC and an indicator for prognostic [30]. It is included as a negative prognostic factor in several existing scores for HCC patients treated by TACE [12, 31]. VI and EHS are also associated with a poor OS [21, 22]. VI increases the risk of portal hypertension and a higher risk of gastrointestinal bleeding and ascites [32]. Although TIPS can relieve this partial portal hypertension, the stent would also simultaneously shunt residual hepatoportal blood flow unblocked by partial portal or hepatic vein tumor thrombus [26]. TACE is recommended as a local therapy to alleviate hepatic lesions in HCC with extrahepatic spread [33]. However, such patients often require targeted therapy, which might cause a significant decrease in intrahepatic arterial diameters and further increase the side effects of TACE on hepatic ischemia in patients after portal shunt [34].

ALT and creatinine are associated with liver and renal functions after TIPS respectively [17, 35]. ALT levels are commonly tested in patients and correlate with hepatic necroinflammation [36], and increased ALT level during treatment is associated with a higher risk of ascites and variceal bleeding, in patients with chronic hepatitis B [37]. Previous prognostic models revealed that high ALT level is a poor prognostic factor of not only pretreatment HCC patients with chronic hepatitis [38] but also post-TIPS patient with cirrhosis [39]. In our study, 88.1% patients were hepatitis B-infected, and elevated ALT levels after TIPS may indicate poor hepatic function for HCC patients who underwent TACE. Serum creatinine has been recognized to be a predictor of prognostic in TIPS-specific model such as FIPS score and MELD score. The C-index, 12- and 24-month AUROC values of FIPS score and MELD score were lower than other TACE-specific models, but the 6-month AUROC values of FIPS score and MELD score are high and close to that of the Pre-TACE-Predict model and HAP score. Therefore, it is possible that the short-term survival of patients treated with TACE after TIPS might have an important relationship with the renal function reserve of TIPS.

There are several limitations. As it is a retrospective study, the risk of selection bias is inherent. The use of TACE in BCLC-C is much less common in western countries. Most patients had HBV-related disease. Although the VACEA score showed a good performance in the subgroup of non-HBV patients, its prognostic ability was overshadowed by the limited numbers in this subgroup. Last, the VACEA score was derived from the baseline characteristics. As these patients may receive systemic treatment, this may weaken its predictive power.

In summary, the VACEA score is a new prognostic model for stratifying recommended TACE candidates with HCC beyond Milan criteria after TIPS. With an easy-to-use presentation consisting of routinely available clinical characters, the model exhibited adequate performance with individualized prediction and can classify patients into four strata with significantly different survival outcomes.

## Supplementary Information

ESM 1 (DOCX 3386 kb)

## Abbreviations

AFP: Alpha-fetoprotein
ALBI: Albumin-bilirubin
ALT: Alanine transaminase
AUROC: Area under receiver operating characteristic curve
BCLC: Barcelona Clinic Liver Cancer
ECOG: Eastern Cooperative Oncology Group
EHS: Extrahepatic spread
HBV: Hepatis B virus
HCC: Hepatocellular carcinoma
mRECIST: Modified Response Evaluation Criteria in Solid Tumors
ORR: Objective response rate
OS: Overall survival
PVTT: Portal vein tumor thrombus
TACE: Transarterial chemoembolization
TIPS: Transjugular intrahepatic portosystemic shunt
VI: Vascular invasion

## Declarations

### Guarantor

The scientific guarantor of this publication is Jiaping Li (Department of Interventional Oncology, The First Affiliated Hospital of Sun Yat-Sen University, Guangzhou, People’s Republic of China).

### Methodology

• retrospective

• prognostic study

• performed at 15 multiple centers
