# Supplementary material for: Identifying optimal candidates for post-TIPS patients with HCC undergoing TACE: a multicenter observational study
Source: Eur Radiol. 2022 Dec 23;33(4):2809–20. doi: 10.1007/s00330-022-09249-6 (PMC10017639; doi:10.1007/s00330-022-09249-6)
Supplement: Supplementary file 1 — (DOCX 3386 kb) [file 330_2022_9249_MOESM1_ESM.docx]

**Supplemental Method**

***TACE Procedures***

A Doppler ultrasonography and liver function test would be performed in patients with TIPS, and TACE would only be performed under full consideration of the patency of the TIPS stent and adequate liver function (Child-Pugh score ≤ 9). Aiming to guarantee the best efficacy for intrahepatic lesions and safety for baseline liver function in HCC patients with TIPS, the gap time between two treatments was less than 4 weeks. TACE included conventional TACE and drug-eluting bead TACE. All procedures were performed by interventional radiologists with ≥7 years of experience. Standard angiographic facilities and protocols were used for hepatic angiography and catheterization. Super-selective catheterizations were performed in every conventional TACE or drug-eluting bead TACE, if possible. However, in patients with bilobar multinodular disease, the lobar artery was selectively catheterized.

For conventional TACE, a solution containing a mixture of doxorubicin (75 mg) or epirubicin (50 mg) with lipiodol was infused; subsequently, either gelatin sponge or polyvinyl alcohol foam particles were introduced until stasis was nearly achieved. The use of drug-eluting bead followed technical recommendations of drug-eluting bead TACE.^1^ DC Bead particles (Biocompatibles, UK) or Callispheres microspheres (Jiangsu Hengrui Medicine Co., Ltd, China) used in most of centers were 100–300 or 300–500 μm in size. Each beads’ vial was loaded with doxorubicin (75 mg) or epirubicin (50 mg). The endpoint of primary chemoembolization was complete HCC devascularization as observed on angiograms [1].


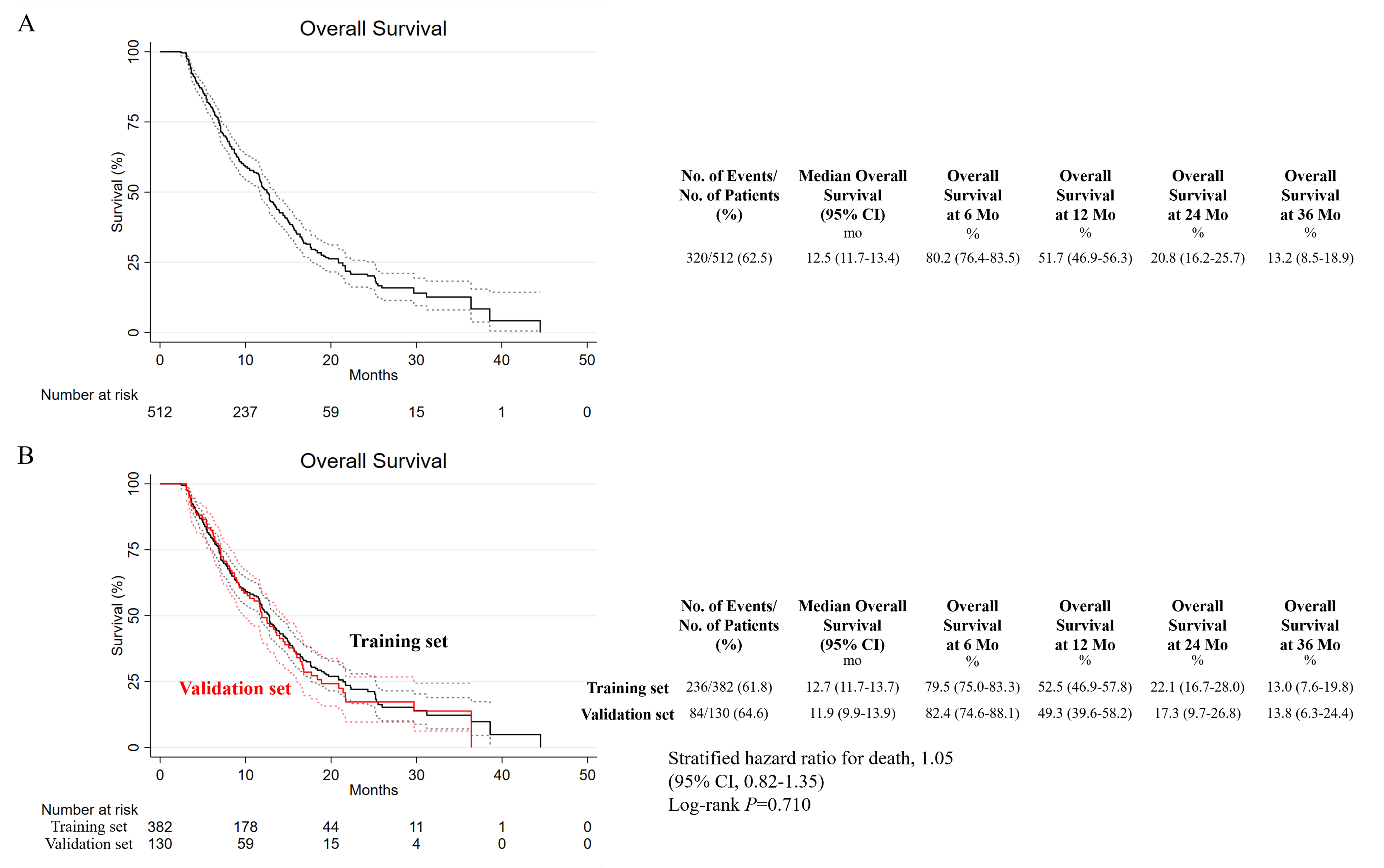


**Supplemental Fig. 1. Overall survival in the total, training and validation sets.**

Overall survival in the (A) total and (B) training and validation sets.


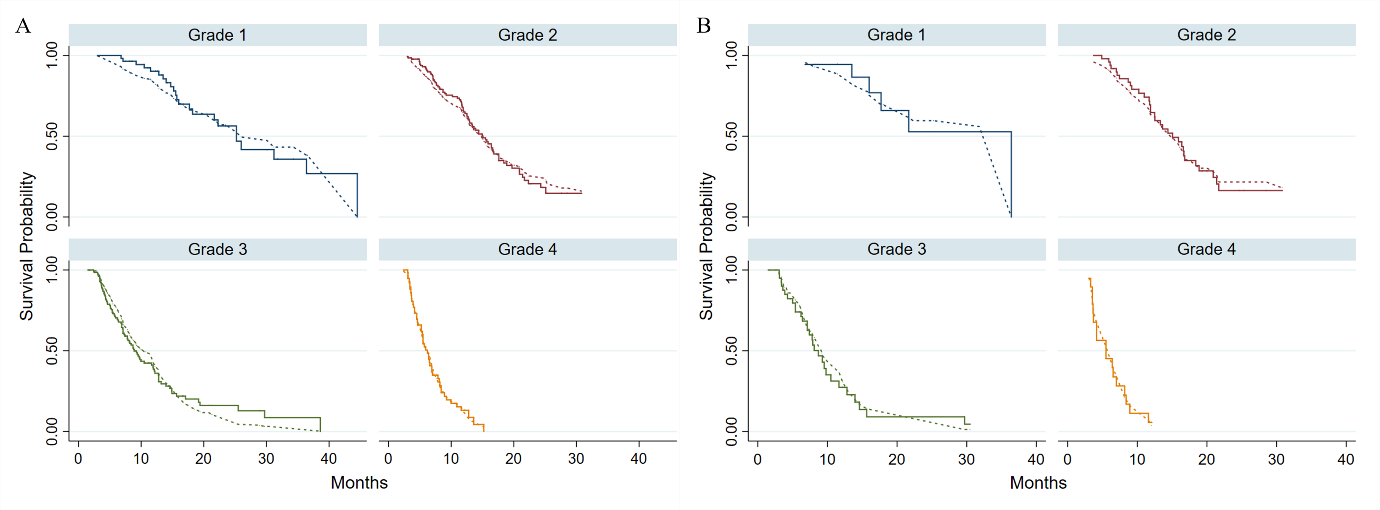


**Supplemental Fig. 2. Calibration of the VACEA model in different risk grades.**

Calibration plot showing the observed (solid line) vs predicted (dashed line) overall survival of different risk grades in (A) training and (B) validation sets.

**Table S1. Previous cohort studies involving TACE plus TIPS**

| **Authors** | **Year** | **Country** | **Sample size** | **Therapy** | **Tumor size (cm)** | **Tumor number 1/>1 (%)** | **Child-Pugh A/B/C, (%)** | **BCLC stage A/B/C, (%)** | **OS** | **TTP** | **ORR (%)** | **DCR (%)** |
| --- | --- | --- | --- | --- | --- | --- | --- | --- | --- | --- | --- | --- |
| Kang JW, et al. [2] | 2012 | Korea | 20 | TACE+TIPS | 3.3 | 65/35 | 35/55/10 | 20/65/15 | 23 months | NR | 70 | 95 |
| Kuo YC, et at. [3] | 2013 | USA | 33 | TACE+TIPS (10) | 2.7 | NR | 50/30/20 | 50/20/0 | 278 days | 103 days | 83 | 100 |
|  |  |  |  | TACE (23) | 2.6 | NR | 35/61/4 | 70/22/0 | 629 days | 232 days | 50 | 90 |
| Kohi MP, et at. [4] | 2013 | USA | 158 | TACE+TIPS (10) | NR | NR | 50/30/20 | 50/20/0 | >400 days | NR | NR | NR |
|  |  |  |  | TACE (148) | NR | NR | 28/60/12 | 52/18/5 | >400 days | NR | NR | NR |
| Miura JT, et al. [5] | 2015 | USA | 16 | TACE+TIPS | 2.8 | 56.2/43.8 | 12.5/75/12.5 | 18.8/25/43.7 | 11.5 months | NR | 56.3 | 93.8 |
| Zhang Y, et al. [6] | 2019 | China | 85 | TACE/TAE+^125^I+TIPS (40) | NR | NR | 15/50/35 | 0/0/100 | 11.8 months | NR | NR | NR |
|  |  |  |  | TACE/TAE+TIPS (45) | NR | NR | 22.2/35.6/42.2 | 0/0/100 | 7.7 months | NR | NR | NR |
| Ruohoniemi DM, et al. [7] | 2020 | USA | 50 | TACE+TIPS (25) | 2.5 | 76/24 | NR | 0/100/0 | >12 months | 283 days | 52.0 | 80.0 |
|  |  |  |  | TACE (25) | 2.2 | 80/20 | NR | 0/100/0 | >12 months | 310 days | 76.0 | 88.0 |
| Fan WZ, et al. [8] | 2021 | China | 119 | DEB-TACE+TIPS (57) | 6.0 | <3: 17.5  ≥3: 82.5 | 68.4/31.6/0 | 7.1/36.8/56.1 | 11.4 months | 6.9 months | 70.2 | 89.5 |
|  |  |  |  | cTACE+TIPS (62) | 8.2 | <3: 11.3  ≥3: 88.7 | 72.6/27.4/0 | 6.5/25.8/67.7 | 9.1 months | 5.2 months | 50.0 | 82.3 |
| Lu HL, et al. [9] | 2021 | China | 103 | TACE/TAE+TIPS (26) | 5.7 | 42.3/57.7 | 15.4/69.2/15.4 | 11.5/38.5/50 | 14 months | NR | 65.4 | NR |
|  |  |  |  | TACE/TAE (77) | 7.1 | 35.1/64.9 | 31.2/53.2/15.6 | 5.2/35.1/59.2 | 9.9 months | NR | 27.4 | NR |

BCLC, Barcelona clinic liver cancer; CP, child-pugh; DCR, disease control rate; NR, not reported; ORR, objective response rate; OS, overall survival; TACE, transarterial chemoembolization; TAE, transarterial embolization; TIPS, transjugular intrahepatic portosystemic; TTP, time to progression.

**Table S2. Previous well recognized prognostic models involving TACE, TIPS, or HCC.**

| **Topic** | **Authors** | **Year** | **Name** | **Candidate** | **Sample size** | **Tumor size (cm)** | **Tumor number 1/>1 (%)** | **CP A/B/C, (%)** | **BCLC stage A/B/C, (%)** | **Prognostic factor** | **Risk grade and OS** |
| --- | --- | --- | --- | --- | --- | --- | --- | --- | --- | --- | --- |
| TIPS | MalinchocM, et al. [10] | 2000 | MELD | - With cirrhosis - Without hepatocellular carcinoma | 231 | NA | NA | 8.1/36.2/55.7 | NA | - log_e_TBil(mg/dL): 0.378 - log_e_INR: 1.120 - log_e_Creatinine(mg/dL): 0.957 - Etiology (0 if cholestatic or alcoholic, 1 otherwise): 0.643 | High risk group: >1.8 point; 2.8 months  Low risk group: ≤1.8 point; 1.3 years |
| TACE | Kadalayil L, et at. [11] | 2013 | HAP | - Any BCLC stage - Solitary tumor >3cm or multifocal | 114 (Training) | ≤5: 54%  >5: 46% | 42/56 | 71/26/3 | 35/31/31 | - ALB <36 g/dl: 1 point - AFP >400ng/ml: 1 point - TBil >17 umol/l: 1 point - Tumor size >7cm: 1 point | HAP A: 0 point; 27.6 months  HAP B: 1 point; 18.5 months  HAP C: 2 points; 9.0 months  HAP D: >2 points; 3.6 months |
|  |  |  |  |  | 166 (Validation) | ≤5: 49%  >5: 51% | 37/63 | 90/10/0 | NR |  |  |
| TACE | Park Y, et al. [12] | 2015 | mHAP-II | - Treatment-naïve - Any BCLC stage   Without EHS | 280 | 3.1 | 50.4/49.6 | 90/10/0 | 55.4/35.0/8.2 | - ALB <36 g/dl: 1 point - AFP >400ng/ml: 1 point - TBil >17 umol/l: 1 point - Tumor size >7cm: 1 point - Tumor number ≥2: 1 point | mHAP-II A: 0 point; 129 months  mHAP-II B: 1 point; 42.7 months  mHAP-II C: 2 points; 33.8 months  mHAP-II D: >2 points; 11.2 months |
| Chironic liver disease | Johnson PJ, et al. [13] | 2015 | ALBI | All disease stages and etiology of chronic liver disease | 1313 (Training) | NR | NR | 65.8/26.6/7.5 | NR | - log_10_TBil(umol/L): 0.66 - ALB (g/L): -0.085 | Grade 1: ≤-2.60; 85.6 months  Grade 2: >-2.60 to ≤-1.39; 46.5 months  Grade 3: >-1.39; 15.5 months |
|  |  |  |  |  | 5097 (Validation) |  |  |  |  |  |  |
| TACE | Li X, et al. [14] | 2015 |  | - Treatment-naïve - Unresectable - Without EHS or CP C | 2938 (Training) | 8.0 | 25.5/74.5 | 95.5/4.5/0 | 9.6/59.2/31.2 | - Portal vein: Branches/ Main - Tumor number: 1/>1 - Tumor capsule - AFP (ng/ml): 0-200-400 - AST (U/L): >40 - ICGR15 | NA |
|  |  |  |  |  | 647 (Internal validation) | 8.2 | 24.9/75.1 | 95.8/4.2/0 | 9.3/61.1/29.6 |  |  |
|  |  |  |  |  | 221 (External validation) | 8.0 | 24.9/75.1 | 95.0/5.0/0 | 8.6/60.2/31.2 |  |  |
| TACE | Cappelli A, et al. [15] | 2016 | mHAP-III | - Treatment-naïve - TACE as first-line treatment - Without VI, EHS or CP C | 361 | 3.5 | 36.8/63.2 | 72.9/27.1/0 | NR | - Tumor number: 0.309 - Tumor size (cm): 0.104 - log_10_AFP (ng/mL): 0.219 - ALB (g/L): -0.405 - TBil (umol/L): 0.151 | NA |
| TACE | Wang QH, et al. [16] | 2019 | Six-and-twelve | - Ideal TACE candidates - BCLC stage A/B - CP score ≤7 | 807 (Training) | 6.1 | 56.6/43.4 | 95.8/4.2/0 | 60.4/39.6 | - Largest tumor diameter (cm) - Tumor number | Stratum 1: ≤6; 49.1 months  Stratum 2: >6 to ≤12; 32.0 months  Stratum 3: >12; 15.8 months |
|  |  |  |  |  | 797 (Validation) | 6.0 | 58.0/42.0 | 94.9/5.1/0 | 62.0/38.0 |  |  |
| TACE | Han GH, et al. [17] | 2020 | Pre-/Post-TACE-Predict | - Treatment-naïve - TACE as first-line treatment - Transplantation - Without EHS | 4921 | NR | 37.0/63.0 | 73.6/24.7/1.7 | NR | Pre   - Tumor number: 0.313 - log_10_tumor size (cm): 1.252 - log_10_AFP (ng/mL): 0.230 - ALB (g/L): -0.0176 - log_10_TBil (umol/L): 0.458 - VI: 0.437 - HBV: 0.149 - Alcohol: 0.333 - Other cause if not HCV/HBV/alcohol: 0.211 | Risk category 1: ≤0.94; 41.02 months  Risk category 2: >0.94 to ≤1.47; 29.18 months  Risk category 3: >1.47 to ≤2.10; 17.99 months  Risk category 4: >2.10; 8.36 months |
|  |  |  |  |  |  |  |  |  |  | Post   - Tumor number: 0.207 - log_10_tumor size (cm): 1.129 - log_10_AFP (ng/mL): 0.147 - log_10_TBil (umol/L): 0.750 - VI: 0.447 - PR: 0.469 - SD:1.143 - PD: 1.354 | Risk category 1: ≤1.82; 55.53 months  Risk category 2: >1.82 to ≤2.49; 30.26 months  Risk category 3: >2.49 to ≤3.37; 17.93 months  Risk category 4: >3.37; 8.36 months |
| TIPS | Bettinger D, et al. [18] | 2021 | FIPS | - refractory ascites - secondary prevention of variceal bleeding | 1496 (Training) | NA | NA | 16.7/65.1/18.2 | NA | - log_10_TBil (umol/ml): 1.43 - 1/Creatinine (mg/dl): 0.219 - Age (year): 0.02 - ALB (g/L): -0.02 | Low risk: <0.92: 48.0 months  High risk: ≥0.92; 5.0 months |
|  |  |  |  |  | 375 (Validation) |  |  | 15.7/66.4/17.9 |  |  |  |

AFP, alpha fetoprotein; ALB, albumin; BCLC, Barcelona clinic liver cancer; CP, child-pugh; EHS, extrahepatic spread; INR, international normalized ratio; NA, not applicable; NR, not reported; TACE, transarterial chemoembolization; TBil, total bilirubin; TIPS, transjugular intrahepatic portosystemic; VI, vascular invasion.

**Table S3. Characteristics of patients in each institute**

| **Baseline characteristics** | **Number/Median** | | | | | | | | | |
| --- | --- | --- | --- | --- | --- | --- | --- | --- | --- | --- |
|  | **Institute 1**  **(n=71)** | | **Institute 2**  **(n=47)** | | **Institute 3**  **(n=43)** | | **Institute 4**  **(n=40)** | | **Institute 5**  **(n=36)** | |
|  | **T set (n=53)** | **V set (n=18)** | **T set (n=35)** | **V set (n=12)** | **T set (n=32)** | **V set (n=11)** | **T set (n=30)** | **V set (n=10)** | **T set (n=27)** | **V set (n=9)** |
| Age (year) | 51 | 49 | 53 | 56 | 52 | 59 | 49 | 51 | 50 | 51 |
| < 50 | 25 | 9 | 11 | 2 | 10 | 3 | 15 | 5 | 13 | 4 |
| ≥ 50 | 28 | 9 | 24 | 10 | 22 | 8 | 15 | 5 | 14 | 5 |
| Gender |  |  |  |  |  |  |  |  |  |  |
| Male | 48 | 15 | 33 | 11 | 31 | 11 | 27 | 10 | 26 | 8 |
| Female | 5 | 3 | 2 | 1 | 1 | 0 | 3 | 0 | 1 | 1 |
| Etiology |  |  |  |  |  |  |  |  |  |  |
| HBV | 47 | 18 | 26 | 8 | 30 | 11 | 27 | 10 | 24 | 9 |
| HCV | 1 | 0 | 3 | 1 | 1 | 0 | 1 | 0 | 2 | 0 |
| Other | 5 | 0 | 6 | 3 | 1 | 0 | 2 | 0 | 1 | 0 |
| TIPS indication |  |  |  |  |  |  |  |  |  |  |
| Secondary prevention of variceal bleeding | 45 | 14 | 28 | 11 | 27 | 10 | 23 | 7 | 20 | 7 |
| Ascites | 8 | 4 | 7 | 1 | 5 | 1 | 7 | 3 | 7 | 2 |
| ECOG score |  |  |  |  |  |  |  |  |  |  |
| 0 | 42 | 16 | 32 | 10 | 23 | 8 | 22 | 7 | 21 | 7 |
| 1 | 11 | 2 | 3 | 2 | 9 | 3 | 8 | 3 | 6 | 2 |
| Platelet count (×10^9^/L) | 167 | 160 | 158 | 132 | 133 | 165 | 160 | 105 | 139 | 142 |
| AFP (ng/ml) | 60.4 | 95.4 | 122.6 | 511.9 | 394.6 | 377.2 | 180.9 | 281.3 | 424.9 | 122.6 |
| < 400 | 34 | 12 | 21 | 6 | 16 | 6 | 16 | 5 | 13 | 4 |
| ≥ 400 | 19 | 6 | 14 | 6 | 16 | 5 | 14 | 5 | 14 | 5 |
| ALT (IU/L) | 51 | 55 | 49 | 47 | 33.5 | 24 | 35.5 | 32.5 | 62 | 66 |
| AST (IU/L) | 58 | 52 | 59 | 49 | 51.5 | 39 | 58 | 56 | 87 | 117 |
| ALB (g/L) | 36.0 | 36.5 | 36.3 | 37.0 | 34.0 | 34.2 | 34.8 | 34.2 | 34.8 | 37.1 |
| TBil (umol/L) | 18.3 | 21.9 | 20.7 | 18.9 | 15.5 | 12.8 | 22.4 | 25.0 | 26.7 | 20.2 |
| Ammonia (umol/L) | 72 | 79 | 59 | 61 | 71 | 71 | 68.5 | 60 | 54.5 | 62 |
| Creatinine (mg/dL) | 0.91 | 0.84 | 0.85 | 0.94 | 0.92 | 0.95 | 0.88 | 0.83 | 0.94 | 0.96 |
| INR | 1.12 | 1.14 | 1.16 | 1.09 | 1.13 | 1.09 | 1.14 | 1.21 | 1.12 | 1.11 |
| Child-Pugh class |  |  |  |  |  |  |  |  |  |  |
| A | 42 | 14 | 28 | 10 | 23 | 11 | 21 | 6 | 19 | 6 |
| B | 11 | 4 | 7 | 2 | 9 | 0 | 9 | 4 | 8 | 3 |
| Intrahepatic tumors number |  |  |  |  |  |  |  |  |  |  |
| Single | 10 | 4 | 6 | 2 | 10 | 2 | 6 | 2 | 3 | 0 |
| Multiple | 43 | 14 | 29 | 10 | 22 | 9 | 24 | 8 | 24 | 9 |
| Main tumor size (cm) | 6.7 | 5.8 | 7.3 | 8.5 | 7.5 | 5.5 | 9.7 | 9.5 | 8.2 | 7.4 |
| Vascular invasion |  |  |  |  |  |  |  |  |  |  |
| No | 16 | 7 | 13 | 4 | 13 | 5 | 8 | 3 | 5 | 2 |
| Yes | 37 | 11 | 22 | 8 | 19 | 6 | 22 | 7 | 22 | 7 |
| Extrahepatic spread |  |  |  |  |  |  |  |  |  |  |
| No | 34 | 12 | 23 | 7 | 25 | 9 | 23 | 8 | 16 | 6 |
| Yes | 19 | 6 | 12 | 5 | 7 | 2 | 7 | 2 | 11 | 3 |
| BCLC stage |  |  |  |  |  |  |  |  |  |  |
| A | 3 | 2 | 2 | 1 | 5 | 1 | 2 | 0 | 0 | 0 |
| B | 10 | 5 | 9 | 2 | 8 | 4 | 4 | 3 | 5 | 2 |
| C | 40 | 11 | 24 | 9 | 19 | 6 | 24 | 7 | 22 | 7 |
| ALBI score | -2.26 | -2.27 | -2.27 | -2.36 | -2.20 | -2.00 | -2.03 | -2.02 | -2.08 | -2.32 |
| FIPS score | 0.83 | 0.74 | 0.83 | 1.01 | 1.16 | 1.24 | 1.12 | 1.01 | 1.08 | 0.97 |
| MELD score | 3.64 | 3.89 | 3.62 | 3.43 | 3.97 | 3.69 | 4.13 | 4.26 | 3.83 | 3.74 |
| Pre-TACE-Predict score | 2.02 | 2.01 | 2.030 | 2.18 | 2.40 | 1.97 | 2.37 | 2.40 | 2.46 | 2.37 |
| HAP score | 2 | 2 | 2 | 2 | 2 | 2 | 2 | 2 | 2 | 2 |
| mHAP-II score | 3 | 3 | 3 | 3 | 3 | 3 | 3 | 3 | 3 | 3 |
| mHAP-III score | -10.40 | -10.14 | -10.10 | -10.44 | -9.58 | -10.60 | -8.18 | -7.90 | -8.33 | -10.27 |
| Median survival time (months) | 12.8 | | 14.4 | | 12.5 | | 11.8 | | 9.4 | |
| **Baseline characteristics** | **Number/Median** | | | | | | | | | |
|  | **Institute 6**  **(n=34)** | | **Institute 7**  **(n=34)** | | **Institute 8**  **(n=31)** | | **Institute 9**  **(n=30)** | | **Institute 10**  **(n=29)** | |
|  | **T set (n=25)** | **V set (n=9)** | **T set (n=25)** | **V set (n=9)** | **T set (n=23)** | **V set (n=8)** | **T set (n=22)** | **V set (n=8)** | **T set (n=22)** | **V set (n=7)** |
| Age (year) | 52 | 50 | 50 | 49 | 48 | 51 | 53 | 56 | 51 | 54 |
| < 50 | 12 | 5 | 12 | 5 | 12 | 4 | 8 | 3 | 8 | 1 |
| ≥ 50 | 13 | 4 | 13 | 4 | 11 | 4 | 14 | 5 | 14 | 6 |
| Gender |  |  |  |  |  |  |  |  |  |  |
| Male | 24 | 8 | 22 | 7 | 20 | 8 | 2 | 0 | 19 | 6 |
| Female | 1 | 1 | 3 | 2 | 3 | 0 | 20 | 8 | 3 | 1 |
| Etiology |  |  |  |  |  |  |  |  |  |  |
| HBV | 22 | 7 | 22 | 7 | 20 | 7 | 19 | 8 | 18 | 6 |
| HCV | 2 | 1 | 0 | 0 | 1 | 1 | 0 | 0 | 0 | 0 |
| Other | 1 | 1 | 3 | 2 | 2 | 0 | 3 | 0 | 4 | 1 |
| TIPS indication |  |  |  |  |  |  |  |  |  |  |
| Secondary prevention of variceal bleeding | 18 | 7 | 20 | 9 | 19 | 5 | 18 | 7 | 18 | 5 |
| Ascites | 7 | 2 | 5 | 0 | 4 | 3 | 4 | 1 | 4 | 2 |
| ECOG score |  |  |  |  |  |  |  |  |  |  |
| 0 | 21 | 8 | 17 | 6 | 15 | 4 | 15 | 5 | 11 | 5 |
| 1 | 4 | 1 | 8 | 3 | 8 | 4 | 7 | 3 | 11 | 2 |
| Platelet count (×10^9^/L) | 164 | 141 | 163 | 147 | 144 | 147 | 172 | 180 | 185 | 152 |
| AFP (ng/ml) | 2543.3 | 3315.7 | 761.6 | 13438 | 79 | 398.1 | 563 | 2546 | 332.2 | 473.5 |
| < 400 | 8 | 3 | 12 | 3 | 14 | 4 | 10 | 3 | 11 | 5 |
| ≥ 400 | 17 | 6 | 13 | 6 | 9 | 4 | 12 | 5 | 11 | 2 |
| ALT (IU/L) | 44.5 | 41.5 | 48.5 | 52 | 35 | 45.5 | 37.5 | 29 | 34.5 | 47.5 |
| AST (IU/L) | 76 | 71 | 81 | 91 | 67 | 88.5 | 78 | 61 | 70.5 | 94 |
| ALB (g/L) | 33.8 | 35.4 | 34.9 | 35.8 | 35.7 | 36.0 | 35.0 | 33.9 | 35.1 | 35.6 |
| TBil (umol/L) | 48.4 | 47.1 | 19.0 | 21.4 | 27.7 | 32.8 | 26.3 | 21.6 | 18.9 | 16.1 |
| Ammonia (umol/L) | 65 | 64 | 65 | 63 | 68 | 56 | 73.5 | 68 | 56.5 | 50 |
| Creatinine (mg/dL) | 0.88 | 0.77 | 1.02 | 1.34 | 0.86 | 0.82 | 0.89 | 0.97 | 0.83 | 0.76 |
| INR | 1.15 | 1.15 | 1.14 | 1.14 | 1.12 | 1.18 | 1.12 | 1.10 | 1.16 | 1.19 |
| Child-Pugh class |  |  |  |  |  |  |  |  |  |  |
| A | 19 | 7 | 17 | 7 | 16 | 5 | 16 | 4 | 16 | 7 |
| B | 6 | 2 | 8 | 2 | 7 | 3 | 6 | 4 | 6 | 0 |
| Intrahepatic tumors number |  |  |  |  |  |  |  |  |  |  |
| Single | 2 | 2 | 3 | 1 | 6 | 1 | 4 | 1 | 5 | 1 |
| Multiple | 23 | 7 | 22 | 8 | 17 | 7 | 18 | 7 | 17 | 6 |
| Main tumor size (cm) | 8.1 | 7.6 | 10.1 | 9.7 | 6.3 | 5.5 | 8.6 | 10.8 | 8.5 | 11.5 |
| Vascular invasion |  |  |  |  |  |  |  |  |  |  |
| No | 7 | 4 | 7 | 3 | 8 | 2 | 6 | 2 | 10 | 3 |
| Yes | 18 | 5 | 18 | 6 | 15 | 6 | 16 | 6 | 12 | 4 |
| Extrahepatic spread |  |  |  |  |  |  |  |  |  |  |
| No | 16 | 4 | 16 | 6 | 17 | 7 | 13 | 4 | 13 | 4 |
| Yes | 9 | 5 | 9 | 3 | 6 | 1 | 9 | 4 | 9 | 3 |
| BCLC stage |  |  |  |  |  |  |  |  |  |  |
| A | 1 | 1 | 1 | 0 | 3 | 0 | 1 | 0 | 3 | 0 |
| B | 4 | 2 | 6 | 3 | 5 | 2 | 5 | 2 | 7 | 4 |
| C | 20 | 6 | 18 | 6 | 15 | 6 | 16 | 6 | 12 | 3 |
| ALBI score | -1.94 | -2.12 | -2.14 | -2.17 | -2.15 | -2.17 | -2.08 | -2.02 | -2.19 | -2.25 |
| FIPS score | 0.81 | 0.66 | 0.98 | 1.33 | 0.87 | 0.89 | 1.13 | 1.32 | 0.66 | 0.56 |
| MELD score | 3.58 | 3.75 | 3.88 | 3.36 | 3.86 | 3.69 | 3.95 | 4.08 | 3.85 | 3.84 |
| Pre-TACE-Predict score | 2.57 | 2.37 | 2.59 | 2.65 | 2.12 | 2.23 | 2.45 | 2.65 | 2.30 | 2.43 |
| HAP score | 2 | 2 | 2 | 2 | 2 | 2 | 2 | 2 | 2 | 2 |
| mHAP-II score | 3 | 3 | 3 | 3 | 3 | 3 | 3 | 3 | 3 | 3 |
| mHAP-III score | -4.56 | -5.47 | -9.28 | -9.15 | -8.82 | -8.20 | -8.46 | -8.44 | -9.59 | -9.96 |
| Median survival time (months) | 13.2 | | 9.2 | | 14.6 | | 14.4 | | 12.0 | |
| **Baseline characteristics** | **Number/Median** | | | | | | | | | |
|  | **Institute 11**  **(n=27)** | | **Institute 12**  **(n=27)** | | **Institute 13**  **(n=24)** | | **Institute 14**  **(n=23)** | | **Institute 15**  **(n=16)** | |
|  | **T set (n=20)** | **V set (n=7)** | **T set (n=21)** | **V set (n=6)** | **T set (n=18)** | **V set (n=6)** | **T set (n=17)** | **V set (n=6)** | **T set (n=12)** | **V set (n=4)** |
| Age (year) | 55 | 49 | 54 | 55 | 50 | 51 | 52 | 48 | 53 | 58 |
| < 50 | 4 | 2 | 7 | 1 | 7 | 2 | 7 | 4 | 4 | 1 |
| ≥ 50 | 16 | 5 | 14 | 5 | 11 | 4 | 10 | 2 | 8 | 3 |
| Gender |  |  |  |  |  |  |  |  |  |  |
| Male | 18 | 6 | 19 | 6 | 16 | 5 | 16 | 6 | 11 | 4 |
| Female | 2 | 1 | 2 | 0 | 2 | 1 | 1 | 0 | 1 | 0 |
| Etiology |  |  |  |  |  |  |  |  |  |  |
| HBV | 18 | 7 | 20 | 6 | 18 | 6 | 16 | 5 | 10 | 3 |
| HCV | 0 | 0 | 0 | 0 | 0 | 0 | 0 | 0 | 0 | 0 |
| Other | 2 | 0 | 1 | 1 | 0 | 0 | 1 | 1 | 2 | 1 |
| TIPS indication |  |  |  |  |  |  |  |  |  |  |
| Secondary prevention of variceal bleeding | 18 | 7 | 17 | 5 | 14 | 3 | 13 | 6 | 10 | 3 |
| Ascites | 2 | 0 | 4 | 1 | 4 | 3 | 4 | 0 | 2 | 1 |
| ECOG score |  |  |  |  |  |  |  |  |  |  |
| 0 | 14 | 4 | 13 | 2 | 12 | 3 | 13 | 5 | 10 | 3 |
| 1 | 6 | 3 | 8 | 4 | 6 | 3 | 4 | 1 | 2 | 1 |
| Platelet count (×10^9^/L) | 165 | 179 | 150 | 136 | 168 | 145 | 126 | 99 | 143 | 168 |
| AFP (ng/ml) | 332.2 | 473.5 | 2169.5 | 1231 | 680.1 | 1012.1 | 381 | 549.8 | 178.3 | 1661.5 |
| < 400 | 10 | 3 | 10 | 3 | 7 | 1 | 9 | 2 | 8 | 2 |
| ≥ 400 | 10 | 4 | 11 | 3 | 11 | 5 | 8 | 4 | 4 | 2 |
| ALT (IU/L) | 55.5 | 52.5 | 41.5 | 51.5 | 47.5 | 30 | 75 | 69 | 47 | 38.5 |
| AST (IU/L) | 60 | 81 | 68 | 81 | 74 | 64 | 62 | 54 | 53 | 60 |
| ALB (g/L) | 35.4 | 35.2 | 35.2 | 36.8 | 33.5 | 33.5 | 36.2 | 34.2 | 32.6 | 35.2 |
| TBil (umol/L) | 28.6 | 16.3 | 23.9 | 22.4 | 20.0 | 19.5 | 21.7 | 26.6 | 26.2 | 30.1 |
| Ammonia (umol/L) | 65.5 | 67 | 65 | 68 | 68 | 62 | 67 | 60 | 64.5 | 65 |
| Creatinine (mg/dL) | 0.87 | 0.90 | 0.85 | 0.94 | 0.81 | 0.84 | 0.87 | 0.93 | 0.96 | 0.76 |
| INR | 1.16 | 1.17 | 1.08 | 1.16 | 1.14 | 1.08 | 1.08 | 1.09 | 1.19 | 1.18 |
| Child-Pugh class |  |  |  |  |  |  |  |  |  |  |
| A | 16 | 6 | 18 | 6 | 11 | 4 | 13 | 5 | 12 | 4 |
| B | 4 | 1 | 3 | 0 | 7 | 2 | 4 | 1 | 0 | 0 |
| Intrahepatic tumors number |  |  |  |  |  |  |  |  |  |  |
| Single | 2 | 1 | 5 | 2 | 2 | 0 | 0 | 0 | 3 | 1 |
| Multiple | 18 | 6 | 16 | 4 | 16 | 6 | 17 | 6 | 9 | 3 |
| Main tumor size (cm) | 7.2 | 5.7 | 7.3 | 9.7 | 8.6 | 7.5 | 7.9 | 8.9 | 7.1 | 6.6 |
| Vascular invasion |  |  |  |  |  |  |  |  |  |  |
| No | 9 | 3 | 8 | 2 | 6 | 3 | 7 | 3 | 6 | 3 |
| Yes | 11 | 4 | 13 | 4 | 12 | 3 | 10 | 3 | 6 | 1 |
| Extrahepatic spread |  |  |  |  |  |  |  |  |  |  |
| No | 15 | 7 | 13 | 4 | 11 | 5 | 13 | 5 | 10 | 3 |
| Yes | 5 | 0 | 8 | 2 | 7 | 1 | 4 | 1 | 2 | 1 |
| BCLC stage |  |  |  |  |  |  |  |  |  |  |
| A | 0 | 0 | 1 | 0 | 1 | 0 | 0 | 0 | 1 | 1 |
| B | 7 | 3 | 5 | 1 | 5 | 3 | 8 | 3 | 5 | 2 |
| C | 13 | 4 | 15 | 5 | 12 | 3 | 9 | 3 | 6 | 1 |
| ALBI score | -2.20 | -2.21 | -2.13 | -2.25 | -2.02 | -2.02 | -2.23 | -1.99 | -1.91 | -2.11 |
| FIPS score | 0.84 | 0.69 | 0.94 | 1.18 | 0.68 | 0.79 | 0.80 | 0.80 | 1.16 | 0.93 |
| MELD score | 3.97 | 4.08 | 3.82 | 4.08 | 3.77 | 3.78 | 3.56 | 3.52 | 4.04 | 3.79 |
| Pre-TACE-Predict score | 2.08 | 1.99 | 2.34 | 2.57 | 2.39 | 2.24 | 2.10 | 2.33 | 2.21 | 2.02 |
| HAP score | 2 | 2 | 2 | 2 | 2 | 2 | 2 | 2 | 2 | 2 |
| mHAP-II score | 3 | 3 | 3 | 3 | 3 | 3 | 3 | 3 | 3 | 3 |
| mHAP-III score | -8.48 | -10.41 | -8.95 | -9.58 | -8.79 | -8.89 | -9.74 | -7.94 | -7.72 | -8.21 |
| Median survival time (months) | 9.5 | | 10.4 | | 16.7 | | 12.1 | | 12.4 | |

T set: training set; V set: validation set.

Institute 1: The first affiliated hospital of Sun Yat-sen university; Institute 2: Nanfang Hospital; Institute 3: The People's Hospital of Guangxi Zhuang Autonomous Region; Institute 4: The First People's Hospital of Yulin; Institute 5: The first affiliated hospital of Guangzhou pharmaceutical university; Institute 6: Guangdong second provincial general hospital; Institute 7: Gaozhou People’s Hospital; Institute 8: Huizhou first hospital; Institute 9: Hainan general hospital; Institute 10: Dongguan people’s hospital; Institute 11: Jiangmen Central Hospital; Institute 12: Jinshazhou Hospital of Guangzhou University of Chinese Medicine; Institute 13: Guangzhou panyu central hospital; Institute 14: Huizhou central people’s hospital; Institute 15: The seventh affiliated hospital of Sun Yat-sen university.

AFP, alpha-fetoprotein; ALB, albumin; ALT, alanine aminotransferase; AST, aspartate aminotransferase; BCLC, Barcelona Clinic Liver Cancer; CI, confidential interval; HBV, hepatitis B virus; PLT, platelet count; PVTT, portal vein tumor thrombus; RBC, red blood cells; TBil, total bilirubin; WBC, white blood cells; INR, international normalized ratio.

**Table S4. Tumor response in the total, training and validation sets.**

| **Variable** | **Group, No (%)** | | |
| --- | --- | --- | --- |
|  | **Total (n=512)** | **Training set (n=382)** | **Validation set (n=130)** |
| Complete response | 81 (15.8) | 57 (14.9) | 24 (18.5) |
| Partial response | 247 (48.2) | 191 (50.0) | 56 (43.1) |
| Stable disease | 100 (19.5) | 74 (19.4) | 26 (20.0) |
| Progressive disease | 84 (16.4) | 60 (15.7) | 24 (18.5) |
| Objective response rate | 328 (64.1) | 248 (64.9) | 80 (61.5) |
| Disease control rate | 428 (83.6) | 322 (84.3) | 106 (81.5) |

**Table S5. Survival outcomes of different risk grades in the training and validation sets.**

| **Cohort** | **Risk grade** | **Median OS (month) (95% CI)** | **HR (95% CI)** | ***P* value** | **6-month survival rate (95% CI)** | **1-year survival rate (95% CI)** | **2-year survival rate (95% CI)** |
| --- | --- | --- | --- | --- | --- | --- | --- |
| Training set | Grade 1 | 25.2 (20.6-29.8) | Ref |  | 100.0 (100.0-100.0) | 90.2 (78.0-95.8) | 56.4 (38.5-70.9) |
|  | Grade 2 | 15.1 (12.6-17.5) | 2.45 (1.51-3.97) | <0.001 | 91.5 (85.1-95.2) | 65.0 (55.6-73.0) | 20.6 (11.9-31.0) |
|  | Grade 3 | 8.9 (7.5-10.3) | 4.62 (2.87-7.45) | <0.001 | 70.4 (61.5-77.6) | 38.6 (29.3-47.8) | 16.1 (8.6-25.7) |
|  | Grade 4 | 6.2 (5.3-7.1) | 11.46 (6.70-19.59) | <0.001 | 50.4 (36.5-62.8) | 13.1 (5.4-24.2) | NA |
| Validation set | Grade 1 | 36.4 (NA) | Ref |  | 100.0 (100.0-100.0) | 94.4 (66.6-99.2) | 52.8 (18.8-78.3) |
|  | Grade 2 | 15.1 (11.6-18.6) | 2.96 (1.15-7.61) | 0.024 | 96.0 (84.8-99.0) | 64.7 (48.8-76.7) | 16.3 (5.8-31.6) |
|  | Grade 3 | 8.7 (6.9-10.5) | 7.62 (2.91-19.94) | <0.001 | 74.1 (57.1-85.1) | 27.3 (12.8-44.1) | 9.1 (1.7-24.5) |
|  | Grade 4 | 5.5 (2.4-8.6) | 21.16 (7.43-60.25) | <0.001 | 45.1 (22.1-65.7) | 5.6 (0.4-22.7) | NA |

CI, confidence interval; HR, hazard ratio; NA, not assessable; Ref, referrence.

**Table S6. Tumor response according to different risk grades in training and validation sets.**

| **Variable** | **Group, No (%)** | | | | | | | | | |
| --- | --- | --- | --- | --- | --- | --- | --- | --- | --- | --- |
|  | **Training set** | | | | | **Validation set** | | | | |
|  | **Grade 1 (n=57)** | **Grade 2 (n=134)** | **Grade 3 (n=133)** | **Grade 4 (n=58)** | ***P* value** | **Grade 1 (n=18)** | **Grade 2 (n=51)** | **Grade 3 (n=42)** | **Grade 4 (n=19)** | ***P* value** |
| Complete response | 22 (38.6) | 22 (16.4) | 11 (8.3) | 2 (3.4) | <0.001 | 8 (44.4) | 12 (23.5) | 3 (7.1) | 1 (5.3) | 0.002 |
| Partial response | 31 (54.4) | 78 (58.2) | 60 (45.1) | 22 (37.9) | 0.034 | 9 (50.0) | 24 (47.1) | 19 (45.2) | 4 (21.1) | 0.210 |
| Stable disease | 3 (5.3) | 19 (14.2) | 37 (27.8) | 15 (25.9) | 0.001 | 1 (5.6) | 6 (11.8) | 12 (28.6) | 7 (36.8) | 0.020 |
| Progressive disease | 1 (1.8) | 15 (11.2) | 25 (18.8) | 19 (32.8) | <0.001 | 0 (0) | 9 (17.6) | 8 (19.0) | 7 (36.8) | 0.039 |
| Objective response rate | 53 (93.0) | 100 (74.6) | 71 (53.4) | 24 (41.4) | <0.001 | 17 (94.4) | 36 (70.6) | 22 (52.4) | 5 (26.3) | <0.001 |
| Disease control rate | 56 (98.2) | 119 (88.8) | 108 (81.2) | 39 (67.2) | <0.001 | 18 (100.0) | 42 (82.4) | 34 (81.0) | 12 (63.2) | 0.002 |

**Table S7. Performance and discriminative ability in the training and validation sets.**

| **Cohort** | **LR χ^2^** | **C-index (95% CI)** | **AIC** |
| --- | --- | --- | --- |
| Training | 115.83 | 0.735 (0.705-0.764) | 2297.973 |
| Validation | 55.25 | 0.771 (0.727-0.816) | 631.091 |

AIC, Akaike Information Criterion; CI, confidence interval; LR, likelihood ratio.

**Table S8. C-indices of the VACEA model and other models across different subgroups in the training cohort.**

| **Subgroup** | **C-index (95% CI)** | | | | | | | |
| --- | --- | --- | --- | --- | --- | --- | --- | --- |
|  | **VACEA** | **Pre-TACE-Predict** | **FIPS** | **MELD** | **ALBI** | **HAP** | **mHAP-II** | **mHAP-III** |
| Age<50 | 0.722 (0.672-0.772) | 0.672 (0.616-0.728) | 0.583 (0.517-0.649) | 0.579 (0.518-0.640) | 0.541 (0.478-0.603) | 0.625 (0.566-0.684) | 0.623 (0.564-0.683) | 0.568 (0.509-0.628) |
| Age≥50 | 0.754 (0.718-0.789) | 0.660 (0.613-0.707) | 0.611 (0.557-0.664) | 0.581 (0.527-0.634) | 0.530 (0.473-0.587) | 0.613 (0.561-0.664) | 0.611 (0.561-0.661) | 0.553 (0.498-0.609) |
| AFP<400 ng/ml | 0.738 (0.692-0.783) | 0.667 (0.611-0.722) | 0.582 (0.522-0.642) | 0.577 (0.519-0.635) | 0.530 (0.466-0.594) | 0.574 (0.517-0.631) | 0.579 (0.519-0.638) | 0.541 (0.480-0.602) |
| AFP≥400 ng/ml | 0.678 (0.629-0.727) | 0.581 (0.528-0.633) | 0.574 (0.518-0.629) | 0.574 (0.518-0.629) | 0.546 (0.487-0.605) | 0.555 (0.496-0.613) | 0.550 (0.494-0.605) | 0.553 (0.495-0.611) |
| HBV | 0.732 (0.700-0.764) | 0.678 (0.642-0.714) | 0.579 (0.535-0.624) | 0.588 (0.546-0.630) | 0.546 (0.502-0.590) | 0.628 (0.588-0.668) | 0.627 (0.587-0.667) | 0.571 (0.529-0.614) |
| Other etiology | 0.763 (0.694-0.831) | 0.582 (0.451-0.714) | 0.638 (0.524-0.751) | 0.586 (0.467-0.704) | 0.480 (0.336-0.625) | 0.586 (0.449-0.722) | 0.556 (0.431-0.682) | 0.484 (0.353-0.615) |
| Secondary prophylaxis of variceal bleeding | 0.714 (0.678-0.750) | 0.656 (0.616-0.697) | 0.596 (0.551-0.641) | 0.598 (0.555-0.641) | 0.531 (0.484-0.578) | 0.602 (0.559-0.645) | 0.600 (0.556-0.643) | 0.555 (0.510-0.600) |
| Ascites | 0.803 (0.756-0.849) | 0.711 (0.640-0.783) | 0.531 (0.433-0.629) | 0.538 (0.434-0.641) | 0.560 (0.462-0.658) | 0.704 (0.622-0.785) | 0.705 (0.628-0.782) | 0.596 (0.499-0.693) |

AFP, alpha-fetoprotein; CI, confidence interval; HBV, hepatitis B virus.

**Table S9. Time-dependent AUROC of the VACEA model and other model across different subgroups in the training cohort.**

| **Subgroup** | **6-month AUROC (95% CI)** | | | | | | | |
| --- | --- | --- | --- | --- | --- | --- | --- | --- |
|  | **VACEA** | **Pre-TACE-Predict** | **FIPS** | **MELD** | **ALBI** | **HAP** | **mHAP-II** | **mHAP-III** |
| Age<50 | 0.817 (0.746-0.888) | 0.703 (0.609-0.797) | 0.734 (0.632-0.836) | 0.669 (0.567-0.765) | 0.622 (0.511-0.733) | 0.670 (0.574-0.766) | 0.653 (0.551-0.755) | 0.636 (0.532-0.740) |
| Age≥50 | 0.818 (0.760-0.876) | 0.665 (0.581-0.749) | 0.651 (0.550-0.752) | 0.631 (0.534-0.728) | 0.524 (0.424-0.624) | 0.628 (0.536-0.720) | 0.634 (0.546-0.722) | 0.552 (0.455-0.649) |
| AFP<400 ng/ml | 0.877 (0.821-0.933) | 0.712 (0.612-0.812) | 0.677 (0.562-0.792) | 0.649 (0.543-0.754) | 0.510 (0.384-0.636) | 0.608 (0.500-0.716) | 0.603 (0.488-0.718) | 0.552 (0.437-0.667) |
| AFP≥400 ng/ml | 0.733 (0.656-0.810) | 0.579 (0.485-0.673) | 0.662 (0.570-0.754) | 0.640 (0.546-0.734) | 0.606 (0.511-0.701) | 0.581 (0.490-0.672) | 0.571 (0.480-0.662) | 0.593 (0.499-0.687) |
| HBV | 0.817 (0.769-0.865) | 0.702 (0.640-0.764) | 0.684 (0.609-0.759) | 0.662 (0.591-0.733) | 0.597 (0.521-0.673) | 0.672 (0.606-0.738) | 0.661 (0.593-0.729) | 0.617 (0.545-0.689) |
| Other etiology | 0.838 (0.722-0.954) | 0.528 (0.260-0.796) | 0.592 (0.402-0.782) | 0.643 (0.468-0.818) | 0.398 (0.141-0.655) | 0.470 (0.196-0.744) | 0.475 (0.219-0.731) | 0.380 (0.219-0.731) |
| Secondary prophylaxis of variceal bleeding | 0.799 (0.746-0.852) | 0.664 (0.593-0.735) | 0.700 (0.625-0.775) | 0.666 (0.590-0.743) | 0.556 (0.472-0.640) | 0.622 (0.547-0.697) | 0.614 (0.538-0.690) | 0.580 (0.500-0.660) |
| Ascites | 0.909 (0.837-0.981) | 0.787 (0.670-0.904) | 0.568 (0.386-0.750) | 0.598 (0.485-0.711) | 0.660 (0.551-0.809) | 0.782 (0.660-0.904) | 0.785 (0.672-0.898) | 0.673 (0.530-0.816) |
| **Subgroup** | **1-year AUROC (95% CI)** | | | | | | | |
|  | **VACEA** | **Pre-TACE-Predict** | **FIPS** | **MELD** | **ALBI** | **HAP** | **mHAP-II** | **mHAP-III** |
| Age<50 | 0.761 (0.690-0.832) | 0.740 (0.651-0.829) | 0.548 (0.449-0.647) | 0.577 (0.478-0.676) | 0.557 (0.458-0.656) | 0.646 (0.555-0.737) | 0.652 (0.560-0.744) | 0.589 (0.492-0.686) |
| Age≥50 | 0.839 (0.778-0.900) | 0.730 (0.654-0.806) | 0.622 (0.538-0.706) | 0.580 (0.495-0.665) | 0.534 (0.448-0.620) | 0.668 (0.588-0.748) | 0.655 (0.576-0.734) | 0.564 (0.478-0.650) |
| AFP<400 ng/ml | 0.791 (0.721-0.861) | 0.709 (0.623-0.795) | 0.565 (0.468-0.662) | 0.564 (0.471-0.657) | 0.504 (0.408-0.600) | 0.567 (0.479-0.655) | 0.576 (0.488-0.664) | 0.519 (0.423-0.615) |
| AFP≥400 ng/ml | 0.724 (0.630-0.818) | 0.626 (0.527-0.725) | 0.573 (0.479-0.667) | 0.573 (0.475-0.670) | 0.595 (0.494-0.696) | 0.575 (0.477-0.673) | 0.564 (0.467-0.661) | 0.603 (0.505-0.701) |
| HBV | 0.794 (0.740-0.848) | 0.746 (0.686-0.806) | 0.568 (0.499-0.637) | 0.590 (0.522-0.658) | 0.560 (0.491-0.629) | 0.665 (0.602-0.728) | 0.664 (0.601-0.727) | 0.594 (0.526-0.662) |
| Other etiology | 0.860 (0.746-0.974) | 0.645 (0.445-0.845) | 0.693 (0.510-0.876) | 0.541 (0.340-0.742) | 0.467 (0.267-0.667) | 0.642 (0.439-0.845) | 0.582 (0.392-0.772) | 0.468 (0.278-0.658) |
| Secondary prophylaxis of variceal bleeding | 0.768 (0.707-0.829) | 0.719 (0.653-0.785) | 0.589 (0.517-0.661) | 0.598 (0.530-0.666) | 0.544 (0.471-0.617) | 0.639 (0.571-0.707) | 0.634 (0.566-0.702) | 0.576 (0.504-0.648) |
| Ascites | 0.948 (0.897-0.999) | 0.799 (0.681-0.917) | 0.531 (0.374-0.688) | 0.527 (0.399-0.654) | 0.540 (0.387-0.693) | 0.745 (0.611-0.879) | 0.747 (0.620-0.874) | 0.588 (0.438-0.738) |
| **Subgroup** | **2-year AUROC (95% CI)** | | | | | | | |
|  | **VACEA** | **Pre-TACE-Predict** | **FIPS** | **MELD** | **ALBI** | **HAP** | **mHAP-II** | **mHAP-III** |
| Age<50 | 0.682 (0.531-0.833) | 0.659 (0.472-0.846) | 0.474 (0.308-0.640) | 0.440 (0.276-0.603) | 0.494 (0.327-0.661) | 0.594 (0.420-0.768) | 0.601 (0.439-0.763) | 0.486 (0.324-0.648) |
| Age≥50 | 0.817 (0.714-0.920) | 0.776 (0.638-0.914) | 0.615 (0.472-0.758) | 0.609 (0.457-0.760) | 0.614 (0.457-0.771) | 0.722 (0.572-0.872) | 0.722 (0.564-0.880) | 0.646 (0.478-0.814) |
| AFP<400 ng/ml | 0.760 (0.650-0.870) | 0.703 (0.569-0.837) | 0.550 (0.419-0.681) | 0.562 (0.416-0.707) | 0.620 (0.494-0.746) | 0.637 (0.501-0.773) | 0.644 (0.504-0.784) | 0.603 (0.463-0.743) |
| AFP≥400 ng/ml | 0.631 (0.490-0.772) | 0.492 (0.340-0.644) | 0.436 (0.180-0.692) | 0.506 (0.305-0.706) | 0.473 (0.220-0.726) | 0.461 (0.341-0.608) | 0.475 (0.321-0.629) | 0.487 (0.274-0.700) |
| HBV | 0.762 (0.666-0.858) | 0.751 (0.627-0.875) | 0.518 (0.400-0.636) | 0.569 (0.434-0.703) | 0.557 (0.440-0.674) | 0.682 (0.560-0.804) | 0.685 (0.565-0.805) | 0.584 (0.457-0.711) |
| Other etiology | 0.884 (0.780-0.988) | 0.537 (0.326-0.748) | 0.731 (0.537-0.925) | 0.661 (0.558-0.764) | 0.950 (0.907-0.993) | 0.678 (0.516-0.840) | 0.679 (0.503-0.855) | 0.821 (0.645-0.997) |
| Secondary prophylaxis of variceal bleeding | 0.798 (0.696-0.900) | 0.719 (0.580-0.858) | 0.562 (0.440-0.684) | 0.563 (0.429-0.698) | 0.558 (0.428-0.688) | 0.645 (0.509-0.781) | 0.645 (0.510-0.780) | 0.576 (0.442-0.710) |
| Ascites | 0.696 (0.497-0.895) | 0.797 (0.613-0.981) | 0.512 (0.188-0.836) | 0.650 (0.489-0.811) | 0.710 (0.470-0.950) | 0.877 (0.771-0.983) | 0.855 (0.733-0.977) | 0.711 (0.460-0.962) |
| **Subgroup** | **3-year AUROC (95% CI)** | | | | | | | |
|  | **VACEA** | **Pre-TACE-Predict** | **FIPS** | **MELD** | **ALBI** | **HAP** | **mHAP-II** | **mHAP-III** |
| Age<50 | 0.839 (0.696-0.982) | 0.756 (0.548-0.964) | 0.535 (0.354-0.716) | 0.447 (0.375-0.518) | 0.606 (0.504-0.708) | 0.702 (0.417-0.987) | 0.684 (0.402-0.966) | 0.544 (0.447-0.640) |
| Age≥50 | 0.701 (0.636-0.766) | 0.691 (0.525-0.857) | 0.727 (0.662-0.792) | 0.708 (0.633-0.783) | 0.651 (0.405-0.897) | 0.614 (0.477-0.751) | 0.647 (0.598-0.696) | 0.653 (0.473-0.833) |
| AFP<400 ng/ml | 0.722 (0.674-0.770) | 0.651 (0.506-0.796) | 0.716 (0.572-0.860) | 0.624 (0.333-0.916) | 0.676 (0.483-0.869) | 0.547 (0.418-0.676) | 0.583 (0.568-0.598) | 0.622 (0.326-0.918) |
| AFP≥400 ng/ml | 0.745 (0.657-0.833) | 0.546 (0.427-0.665) | 0.370 (0.234-0.506) | 0.491 (0.324-0.657) | 0.483 (0.126-0.840) | 0.494 (0.255-0.733) | 0.476 (0.260-0.692) | 0.470 (0.277-0.663) |
| HBV | 0.812 (0.636-0.988) | 0.756 (0.534-0.978) | 0.641 (0.387-0.895) | 0.618 (0.325-0.910) | 0.606 (0.457-0.755) | 0.667 (0.460-0.874) | 0.677 (0.457-0.897) | 0.606 (0.368-0.844) |
| Other etiology | NA | NA | NA | NA | NA | NA | NA | NA |
| Secondary prophylaxis of variceal bleeding | 0.837 (0.687-0.987) | 0.740 (0.513-0.967) | 0.632 (0.359-0.905) | 0.579 (0.326-0.832) | 0.595 (0.510-0.680) | 0.648 (0.420-0.876) | 0.654 (0.401-0.907) | 0.578 (0.383-0.773) |
| Ascites | 0.724 (0.488-0.960) | 0.725 (0.567-0.883) | 0.907 (0.824-0.990) | 0.935 (0.880-0.990) | 0.834 (0.764-0.904) | 0.821 (0.766-0.876) | 0.799 (0.744-0.854) | 0.843 (0.792-0.894) |

AFP, alpha-fetoprotein; AUROC, area under receiver operating characteristic curve; CI, confidence interval; HBV, hepatitis B virus; NA, not accessable.

**Referrence for Supplemental Material**

1. Lencioni R, de Baere T, Burrel M, et al. Transcatheter treatment of hepatocellular carcinoma with doxorubicin-loaded DC Bead (DEBDOX): technical recommendations. Cardiovasc Intervent Radiol 2012;35:980–985
2. Kang JW, Kim JH, Ko GY, et al. Transarterial chemoembolization for hepatocellular carcinoma after transjugular intrahepatic portosystemic shunt. Acta Radiol. 2012;53(5):545-50.
3. Kuo YC, Kohi MP, Naeger DM, et al. Efficacy of TACE in TIPS patients: comparison of treatment response to chemoembolization for hepatocellular carcinoma in patients with and without a transjugular intrahepatic portosystemic shunt. Cardiovasc Intervent Radiol. 2013;36(5):1336-43.
4. Kohi MP, Fidelman N, Naeger DM, et al. Hepatotoxicity after transarterial chemoembolization and transjugular intrahepatic portosystemic shunt: do two rights make a wrong? J Vasc Interv Radiol. 2013;24(1):68-73.
5. Miura JT, Rilling WS, White SB, et al. Safety and efficacy of transarterial chemoembolization in patients with transjugular intrahepatic portosystemic shunts. HPB (Oxford). 2015;17(8):707-12.
6. Zhang Y, Wu YF, Yue ZD, et al. Iodine-125 implantation with transjugular intrahepatic portosystemic shunt for main portal vein tumor thrombus. World J Gastrointest Oncol. 2019;11(4):310-321.
7. Ruohoniemi DM, Taslakian B, Aaltonen EA, et al. Comparative Analysis of Safety and Efficacy of Transarterial Chemoembolization for the Treatment of Hepatocellular Carcinoma in Patients with and without Pre-Existing Transjugular Intrahepatic Portosystemic Shunts. J Vasc Interv Radiol. 2020;31(3):409-415.
8. Fan W, Guo J, Zhu B, et al. Drug-eluting beads TACE is safe and non-inferior to conventional TACE in HCC patients with TIPS. Eur Radiol. 2021;31(11):8291-8301.
9. Lu HL, Xuan FF, Luo YC, et al. Efficacy and safety of transjugular intrahepatic portosystemic shunt combined with transcatheter embolization/chemoembolization in hepatocellular carcinoma with portal hypertension and arterioportal shunt. Abdom Radiol (NY). 2021;46(11):5417-5427.
10. Malinchoc M, Kamath PS, Gordon FD, et al. A model to predict poor survival in patients undergoing transjugular intrahepatic portosystemic shunts. Hepatology. 2000 Apr;31(4):864-71. doi: 10.1053/he.2000.5852
11. Kadalayil L, Benini R, Pallan L, et al. A simple prognostic scoring system for patients receiving transarterial embolisation for hepatocellular cancer. Ann Oncol. 2013;24(10):2565-2570.
12. Park Y, Kim SU, Kim BK, et al. Addition of tumor multiplicity improves the prognostic performance of the hepatoma arterial-embolization prognostic score. Liver Int. 2016;36(1):100-7.
13. Johnson PJ, Berhane S, Kagebayashi C, et al. Assessment of liver function in patients with hepatocellular carcinoma: a new evidence-based approach-the ALBI grade. J Clin Oncol. 2015;33(6):550-8.
14. Xu L, Peng ZW, Chen MS, et al. Prognostic nomogram for patients with unresectable hepatocellular carcinoma after transcatheter arterial chemoembolization. J Hepatol. 2015;63(1):122-30.
15. Cappelli A, Cucchetti A, Cabibbo G, et al. Refining prognosis after trans-arterial chemo-embolization for hepatocellular carcinoma. Liver Int. 2016;36(5):729-36.
16. Wang Q, Xia D, Bai W, et al. Development of a prognostic score for recommended TACE candidates with hepatocellular carcinoma: A multicentre observational study. J Hepatol. 2019;70(5):893-903.
17. Han G, Berhane S, Toyoda H, et al. Prediction of Survival Among Patients Receiving Transarterial Chemoembolization for Hepatocellular Carcinoma: A Response-Based Approach. Hepatology. 2020;72(1):198-212.
18. Bettinger D, Sturm L, Pfaff L, et al. Refining prediction of survival after TIPS with the novel Freiburg index of post-TIPS survival. J Hepatol. 2021;74(6):1362-1372.
